# First total synthesis of kipukasin A

**DOI:** 10.3762/bjoc.13.86

**Published:** 2017-05-09

**Authors:** Chuang Li, Haixin Ding, Zhizhong Ruan, Yirong Zhou, Qiang Xiao

**Affiliations:** 1 Jiangxi Key Laboratory of Organic Chemistry, Jiangxi Science & Technology Normal University, Nanchang, Jiangxi 330013, China

**Keywords:** gold catalysis, kipukasin A, marine nucleoside, total synthesis, Vorbrüggen glycosylation

## Abstract

In this paper, a practical approach for the total synthesis of kipukasin A is presented with 22% overall yield by using tetra-*O*-acetyl-β-D-ribose as starting material. An improved iodine-promoted acetonide-forming reaction was developed to access 1,2-*O*-isopropylidene-α-D-ribofuranose. For the first time, *ortho-*alkynylbenzoate was used as protecting group for the 5-hydoxy group. After subsequent Vorbrüggen glycosylation, the protecting group could be removed smoothly in the presence of 5 mol % Ph_3_PAuOTf in dichloromethane to provide kipukasin A in high yield and regioselectivity.

## Introduction

Endogenous nucleosides are involved in DNA and RNA synthesis, cell signalling, enzyme regulation and metabolism etc. [1–2]. Therefore, the synthesis of novel nucleosides to mimic their physiological counterparts has potential therapeutic significance, which has led to the development of a large number of antiviral and antitumor drugs [3–4]. On the other hand, naturally occurring nucleosides, especially marine nucleosides, have also played an indispensable role in drug discovery, which make great contribution in the commercialization of cytosine arabinoside (Ara-C), adenine arabinoside (Ara-A) and AZT, etc. [5–6]. Nucleosides and their analogues will continue to play an important role in future drug discovery [7].

**Figure 1:**
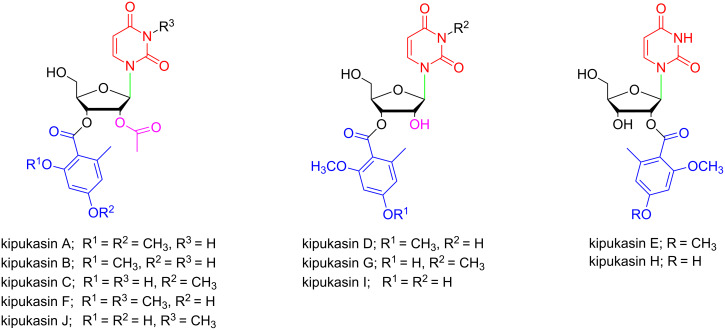
Structures of kipukasins A–J.

In the past decades, exploration of novel naturally occurring marine nucleosides has made expeditious achievements [8–10]. Some of them showed promising antibiotic, antiviral, antiparasitic and antitumor properties. Kipukasins A–G were firstly isolated from solid-substrate fermentation cultures of Hawaiian *Aspergillus Versicolor* in 2007 (Figure 1) [11]. Later on, kipukasins H, I [12] and J [13] were also isolated from the fungus *Aspergillus flavus*, which was collected at the South China Sea and the Sea of Okhotsk, respectively.

Kipukasins are uridine derivatives with unique structural characteristics, which include: (1) a uracil moiety with or without an *N*-3 methyl group; (2) a 6-methyl-2,4-hydroxy (or methoxy)-benzoyl group at C-2’ or C-3’ position; (3) with or without an acetyl group at 2’-OH position. To the best of our knowledge, they are the first naturally occurring aroyl nucleosides reported up to now. The biological assays showed that kipukasin A owned modest activity against Gram-positive bacteria *Staphylococcus aureus* (ATCC 29213) [11].

During our ongoing biological studies of marine nucleosides, total syntheses of several marine nucleosides were accomplished in our group [14–18]. In the present paper, we reported a practical approach for the total synthesis of kipukasin A.

## Results and Discussion

**Figure 2:**
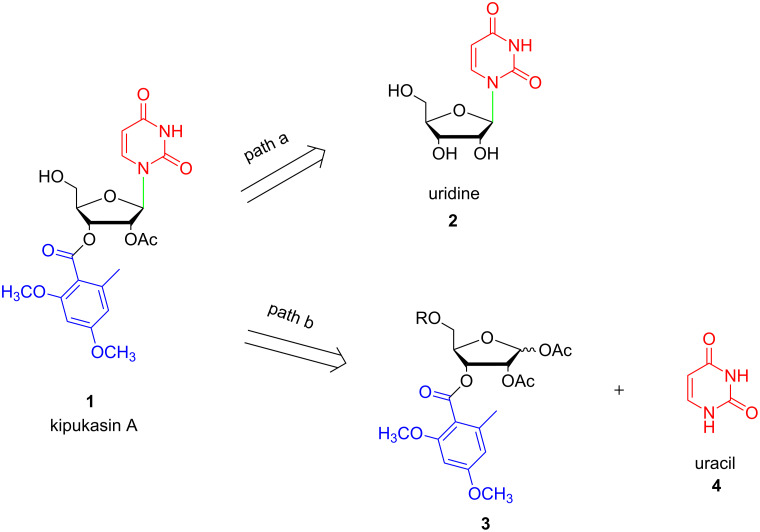
Retrosynthetic analysis of kipukasin A.

From the synthetic point of view, it seemed that the most direct approach for the synthesis of kipukasin A was the regioselective modification of commercially available uridine (Figure 2, path a). After carefully assessment, we realized that it would require several steps of protection and deprotection. Especially under alkaline conditions, 2’,3’-transesterification is inevitable to occur in nucleosides [19–21]. The synthetic route would be lengthy and cumbersome. Therefore, a practical total synthesis is in high demand to facilitate the preparation of other kipukasins and their analogues.

The retrosynthetic analysis is shown in Figure 2 (path b). Kipukasin A could be constructed by Vorbrüggen glycosylation [22–23] of a properly protected glycosyl donor **3** with uracil (**4**). Neighboring group participation of the 2’-*O*-acetyl group stereoselectively facilitate the β-glycosidic bond formation. Thus, the choice of a suitable protecting group at 5-OH position would be crucial for the success. It should fulfill at least two requirements: (1) it should be stable during the Vorbrüggen glycosylation; and (2) the deprotection process should be performed under very mild and neutral conditions without any influence on the 2’-*O*-acetyl group. At the same time, ester protection is preferred for Vorbrüggen glycosylations in nucleoside syntheses. Very recently, *ortho-*alkynylbenzoate was successfully developed by our group as neighboring participation group to synthesize 2’-modified nucleosides [24], which could be removed smoothly in the presence of gold(I) complexes with high yield and selectivity. The conditions are very mild and neutral. In the present paper, we continue to use *ortho-*alkynylbenzoate as protecting group for the 5’-OH group to fulfill the total synthesis of kipukasin A.

**Scheme 1:**
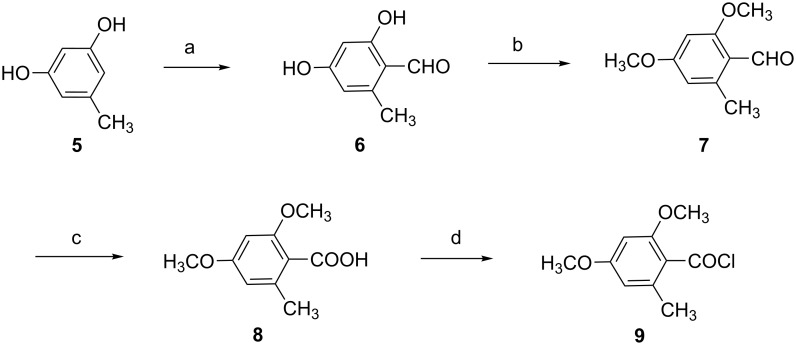
Synthesis of 2,4-dimethoxy-6-methylbenzoic chloride. Reagents and conditions: (a) POCl_3_, DMF, 0 °C to rt, 75%; (b) MeI, K_2_CO_3_, acetone, rt, 93%; (c) NaClO_2_, NaH_2_PO_4_, DMSO, rt, 81%; (d) (COCl)_2_, CH_2_Cl_2_, refux.

According to the retrosynthetic analysis, we firstly started to synthesis of aroyl building block **9** (Scheme 1). Vilsmeier formylation of 1,3-dihydroxy-5-methylbenzene (**5**) gave 2,4-dihydroxy-6-methylbenzaldehyde (**6**) in 75% yield [25–26]. Then compound **6** could react with methyl iodine in acetone by using K_2_CO_3_ as base. The obtained 2,4-dimethoxy-6-methylbenzaldehyde (**7**) was further oxidized with NaH_2_PO_4_/NaClO_2_ in DMSO to provide 2,4-dimethoxy-6-methylbenzoic acid (**8**) in 81% yield [27–28]. Finally, 2,4-dimethoxy-6-methylbenzoyl chloride (**9**) was obtained by refluxing with oxalyl chloride in dichloromethane. After removing the solvent and excess oxalyl chloride, 2,4-dimethoxy-6-methylbenzoyl chloride (**9**) was used directly in the next step without further purification.

**Scheme 2:**
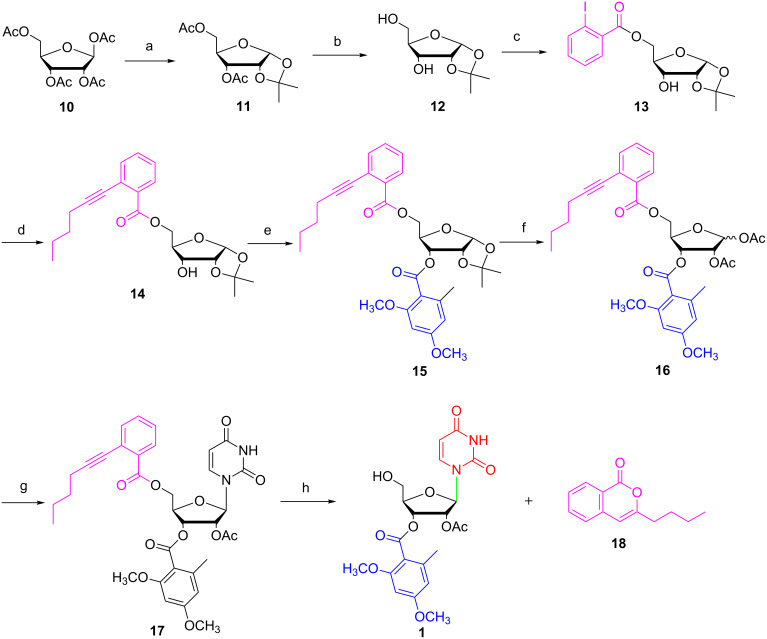
Total synthesis of kipukasin A. Reagents and conditions: (a) I_2_, acetone, 0 °C to rt, 88%; (b) K_2_CO_3_, MeOH, rt, 93%; (c) 2-iodobenzoyl chloride, pyridine, −10 °C to rt, CH_2_Cl_2_, 80%; (d) 1-hexyne, PdCl_2_(PPh_3_)_3_, CuI, Et_3_N, THF, 50 °C , 78%; (e) **9**, DMAP, Et_3_N, CH_2_Cl_2_, 0 °C to rt, 74%; (f) Ac_2_O, H_2_SO_4_, acetic acid, rt, 74%; (g) uracil, BSA, TMSOTf, MeCN, 75 °C, 89%; (h) 5% Ph_3_PAuOTf, H_2_O, CH_2_Cl_2_, EtOH, rt, 90%.

**Figure 3:**
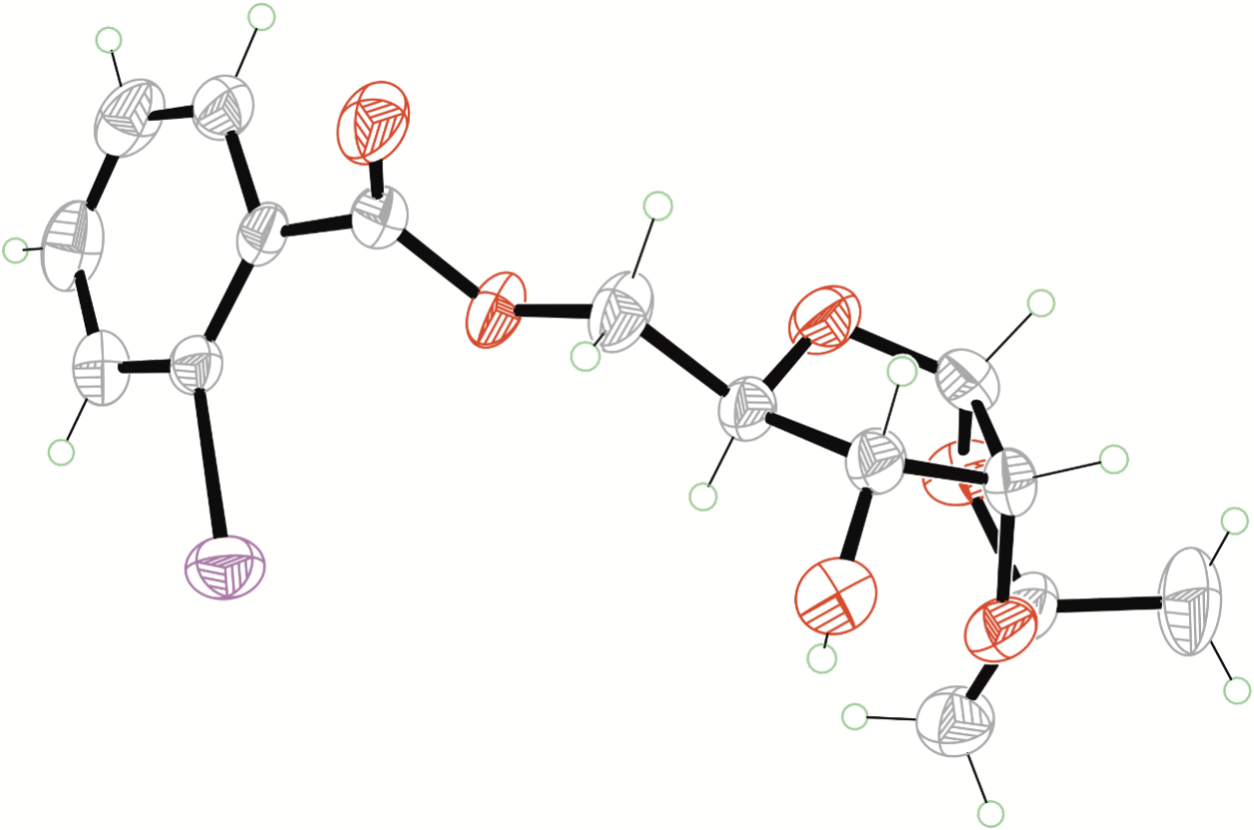
X-ray structure of compound **13**.

Then we started to synthesize glycosylation donor **16** as the key building block (Scheme 2). In previous reports, 3,5-*O*-diacetyl-1,2-*O*-isopropylidene-D-ribofuranose (**11**) was prepared either from D-xylose [29–31] or from tetra-*O*-acetyl-β-D-ribose (**10**) [32–33]. In 2009, Koreeda reported an iodine-promoted acetonide-forming reaction of tetra-*O*-acetyl-β-D-ribose (**10**) [33]. In this preliminary paper, 25 mol % of iodine was necessary. After systematic optimization, it was found that 6 mol % iodine could complete the reaction efficiently in freshly dried acetone to give 3,5-*O*-diacetyl-1,2-*O*-isopropylidene-D-ribofuranose (**11**) in 88% yield. Then cleavage of the remaining acetyl groups by K_2_CO_3_ in MeOH afforded 1,2-*O*-isopropylidene-D-ribofuranose (**12**) in 93% yield. Subsequently, the reaction of 1,2-*O*-isopropylidene-D-ribofuranose (**12**) with 2-iodobenzoyl chloride (0.9 equiv) gave the corresponding 5-*O*-benzoyl ester **13** in 80% yield along with a small amount of the 3,5-dibenzoyl ester. The structure of 5-*O*-benzoyl ester **13** was unambiguously confirmed by X-ray diffraction analysis (Figure 3) [34]. Then Sonogashira cross-coupling with 1-hexyne provided ribose **14** in 78% yield [35].

Subsequently, using DMAP as acylation catalyst and triethylamine as base, the former synthesized 2,4-dimethoxy-6-methylbenzoyl chloride (**9**) reacted with ribose **14** to 3-*O*-(2,4-dimethoxy-6-methylbenzoyl)ribose **15** in 74% yield. Various spectral analyses (NMR, HPLC) showed no evidence that 2,3-*O*-transesterification occurred during the esterification reaction. After cleavage of the acetonide group with acetic acid/acetic anhydride/H_2_SO_4_, the key glycosylation donor **16** was obtained in 74% yield as a mixture of isomers (α/β = 1:8) [36].

With glycosylation donor **16** in hand, we proceeded to investigate the crucial Vorbrüggen glycosylation with uracil (**4**). To our delight, in a similar manner as our described in [17], it proved to be efficient to give nucleoside **17** with exclusive β-configuration in 89% yield. At last, using our developed approach [24], kipukasin A was obtained in 90% yield in the presence of 5 mol % Ph_3_PAuOTf in dichloromethane with H_2_O (1 equiv) and ethanol (6 equiv). All spectra of the synthetic kipukasin A were consistent with an authentic sample.

## Conclusion

In summary, the first total synthesis of kipukasin A was accomplished with 22% overall yield. The reaction sequence includes: (1) an improved iodine-promoted acetonide-forming reaction to synthesize 1,2-*O*-isopropylidene-D-ribofuranose (**12**); (2) a Vorbrüggen glycosylation facilitating the preparation for kipukasin derivatives and (3) the first use of *ortho-*alkynylbenzoate as protecting group of the 5-hydoxy group, which can be removed smoothly in the presence of 5 mol % Ph_3_PAuOTf in dichloromethane. Biological studies of kipukasin A and the total synthesis of other kipukasin nucleosides by this established approach are ongoing in our group.

## Experimental

All reagents and catalysts were purchased from commercial sources (Acros or Aldrich) and used without purification. DCM and CH_3_CN were dried over CaH_2_ and distilled prior to use. Et_3_N was dried over NaH and distilled prior to use. Thin-layer chromatography was performed using silica gel GF-254 plates with detection by UV (254 nm) or charting with 10% sulfuric acid in ethanol. Column chromatography was performed on silica gel (200–300 mesh, Qing-Dao Chemical Company, China). NMR spectra were recorded on a Bruker AV400 spectrometer, and chemical shifts (δ) are reported in ppm. ^1^H NMR and ^13^C NMR spectra were calibrated with TMS as internal standard, and coupling constants (*J*) are reported in Hz. The ESI-HRMS were obtained on a AB SCIEX Triple TOF 4600 spectrometer in positive ion mode. Melting points were measured on an electrothermal apparatus and are uncorrected. Optical rotation values were measured with a Rudolphautopol IV polarimeter.

**Synthesis of 3,5-*O*-diacetyl-1,2-*O*-isopropylidene-D-ribofuranose (11):** To a solution of 1,2,3,5-*O*-acetyl-β-D-ribofuranose (**10**, 10.0 g, 31.4 mmol) in dry acetone (100 mL) was added I_2_ (0.5 g, 2.0 mmol) at 0 °C under argon. After addition, the solution was stirred for 5 h at room temperature and quenched with 40 mL Na_2_S_2_O_3_ (3.0 g, 19.0 mmol). The solvent was evaporated under reduced pressure and distilled water (200 mL) was added to the residue. After that, the solution was extracted with CH_2_Cl_2_ (100 mL × 3), the combined organic layer was washed with sat. aq NaHCO_3_ (100 mL), brine (100 mL), and dried with anhydrous Na_2_SO_4_. After filtration, the filtrate was evaporated under reduced pressure. The residue was purified by column chromatography (silica gel, PE/EtOAc 1:2, v:v) to afford **11** as a light-yellow oil (7.6 g, 88%). [α]^D^_25_ +133.3 (*c* 0.1, acetone) (lit. [37] [α]^D^_25_ +125.9 (*c* 1.1, CHCl_3_)); ^1^H NMR (400 MHz, DMSO-*d*_6_) δ 5.81 (d, *J* = 3.7 Hz, 1H), 4.77 (t, *J* = 4.2 Hz, 1H), 4.67 (dd, *J* = 9.1, 4.8 Hz, 1H), 4.24 (dd, *J* = 12.1, 2.7 Hz, 1H), 4.20–4.15 (m, 1H), 4.05 (dd, *J* = 12.1, 5.4 Hz, 1H), 2.07 (s, 3H), 2.03 (s, 3H), 1.45 (s, 3H), 1.27 (s, 3H); ^13^C NMR (101 MHz, DMSO-*d*_6_) δ 170.1, 169.7, 112.2, 103.9, 76.8, 75.1, 71.9, 62.4, 26.4, 20.5, 20.4; LRMS (ESI) *m/z*: 297.2 [M + Na]^+^; HRMS (ESI) *m/z*: [M + Na]^+^ calcd for C_12_H_18_O_7_Na, 297.0945; found, 297.0943.

**Synthesis of 1,2-*O*-isopropylidene-α-D-ribofuranose (12):** The light-yellow oil **11** (7.60 g, 27.7 mmol) was dissolved in MeOH (60 mL). To the solution K_2_CO_3_ (0.60 g, 4.4 mmol) was added and the reaction mixture was stirred for 2 h at room temperature. The solvent was evaporated under reduced pressure and the residue was purified by silica gel column to give **12** as a white solid (4.60 g, 93%). R*_f_* 0.30 (CH_2_Cl_2_/CH_3_OH 10:1, v:v); mp 90–91 °C (lit. [38] 87–89 °C); [α]^D^_25_ +62.3 (*c* 0.1, CH_3_OH) (lit. [38] [α]^D^_25_ +49 (*c* 0.94, CHCl_3_)); ^1^H NMR (400 MHz, DMSO-*d*_6_) δ 5.65 (d, *J* = 3.7 Hz, 1H), 4.98 (d, *J* = 6.7 Hz, 1H), 4.64 (t, *J* = 5.6 Hz, 1H), 4.43 (t, *J* = 3.9 Hz, 1H), 3.79–3.56 (m, 3H), 3.41–3.35 (m, 1H), 1.43 (s, 3H), 1.26 (s, 3H); ^13^C NMR (101 MHz, DMSO-*d*_6_) δ 111.1, 103.3, 80.3, 79.1, 70.5, 60.2, 26.6, 26.4; LRMS (ESI) *m/z*: 213.3 [M + Na]^+^, 189.3 [M − H]^−^; HRMS (ESI) *m/z*: [M + Na]^+^ calcd for C_8_H_13_O_5_Na, 213.0733; found, 213.0731.

**Synthesis of 1,2-*O*-isopropylidene-5-*O*-(2-iodobenzoyl)-α-D-ribofuranose (13):** To a solution of **12** (6.60 g, 34.7 mmol) in dry CH_2_Cl_2_ (50 mL) and dry pyridine (7.62 mL) were added 0.2 mL 2-iodobenzoyl chloride (8.4 g, 31.5 mmol) at −10 °C under argon. After addition, the reaction mixture was stirred overnight and quenched with iced water (5 mL). The mixture was washed with sat. NaHCO_3_ (40 mL), brine (40 mL), and dried over anhydrous MgSO_4_. After filtration, the filtrated was evaporated to dryness under reduced pressure. The remaining residue was recrystallized by ethanol to obtain **13** as a white powder solid (10.7 g, 80%). R*_f_* 0.35 (PE/EtOAc 2:1, v:v); mp 126–127 °C; [α]^D^_25_ +26.86 (*c* 0.18, CH_3_OH); ^1^H NMR (400 MHz, CDCl_3_) δ 7.99 (d, *J* = 8.0 Hz, 1H), 7.82 (dd, *J* = 7.8, 1.6 Hz, 1H), 7.39 (t, *J* = 7.6 Hz, 1H), 7.15 (td, *J* = 7.7 , 1.6 Hz,1H), 5.84 (d, *J* = 3.8 Hz, 1H), 4.70 (dd, *J* = 12.3, 2.5 Hz, 1H), 4.61 (t, *J* = 4.4 Hz 1H), 4.45 (dd, *J* = 12.3, 5.2 Hz, 1H), 4.11–4.07 (m, 1H), 3.98 (dd, *J* = 9.0, 5.1 Hz, 1H), 2.23 (brs, 1H), 1.58 (s, 3H), 1.37 (s, 3H); ^13^C NMR (101 MHz, CDCl_3_) δ 166.4, 141.4, 134.8, 133.0, 131.4, 128.0, 113.0, 104.2, 94.3, 78.4, 78.3, 72.2, 64.1, 26.7, 26.6; HRMS (ESI) *m/z*: [M + Na]^+^ calcd for C_15_H_17_IO_6_Na, 442.9967; found, 442.9980.

**Synthesis of 1,2-*O*-isopropylidene-5-*O*-(2-(hex-1-yn-1-yl)benzoyl)-α-D-ribofuranose (14):** To a solution of **13** (9.0 g, 21.4 mmol) in dry Et_3_N (25 mL) and dry THF (50 mL) was added CuI (0.41 g, 2.1 mmol), PdCl_2_(PPh_3_)_3_ (2.07 g, 2.1 mmol) and 1-hexyne (2.68 mL, 23.5 mmol). After addition, the reaction mixture was heated at 50 °C for 1 h. TLC detection showed the reaction was finished. The reaction mixture was filtered over a bed of celite. After filtration, the filtrate was evaporated under reduced pressure. The obtained residue was purified by silica gel column chromatography (PE/EtOAc 3:1, v:v) to afford **14** as deep green oil (6.35 g, 78%). R*_f_* 0.43 (PE/EtOAc 2:1,v:v); [α]^D^_25_ +21.33 (*c* 0.15, CH_3_OH); ^1^H NMR (400 MHz, DMSO-*d*_6_) δ 7.81 (d, *J* = 7.8 Hz, 1H), 7.90–7.58 (m, 2H), 7.45 (td, *J* = 7.2, 2.0 Hz, 1H ), 5.71 (d, *J* = 3.6 Hz, 1H), 5.34 (d, *J* = 6.9 Hz, 1H), 4.56–4.50 (m, 2H), 4.22 (dd, *J* = 12.2, 6.1 Hz, 1H), 4.05–4.01 (m, 1H), 3.86–3.81 (m, 1H), 2.45 (t, *J* = 6.9 Hz, 2H), 1.55–1.50 (m, 2H), 1.47–1.41 (m, 5H), 1.27 (s, 3H), 0.91 (t, *J* = 7.2 Hz, 3H); ^13^C NMR (101 MHz, DMSO-*d*_6_) δ 165.6, 133.8, 132.0, 131.9, 129.8, 127.8, 123.3, 111.5, 103.5, 95.9, 78.9, 78.8, 76.9, 71.3, 64.1, 30.1, 26.6, 26.3, 21.4, 18.6, 13.5; HRMS (ESI) *m/z*: [M + Na]^+^ calcd for C_21_H_26_O_6_Na, 397.1627; found, 397.1608.

**Synthesis of 1,2-*O*-isopropylidene-3-*O*-(2,4-dimethoxy-6-methylbenzoyl)-5-*O*-(2-(hex-1-yn-1-yl)benzoyl)-α-D-ribofuranose (15):** To a solution of **14** (3.0 g, 8.0 mmol) in dry CH_2_Cl_2_ (25 mL) was added DMAP (97.88 mg, 0.8 mmol) and Et_3_N (1.05 g, 10.4 mmol). To the mixture benzoyl chloride **9** (2.15 g, 10 mmol) in dry CH_2_Cl_2_ (10 mL) was slowly added at 0 °C and stirred overnight at room temperature. The reaction was quenched with methanol (5 mL) and evaporated to dryness under reduced pressure. The obtained residue was dissolved in CH_2_Cl_2_ (40 mL), washed with sat*.* NaHCO_3_ (40 mL × 2), brine (30 mL × 2), and dried over anhydrous MgSO_4_. The obtained residue was purified by a silica gel column chromatography (PE/EtOAc 4:1, v:v) to afford **15** as colorless oil (3.3 g, 74%). R*_f_* 0.46 (PE/EtOAc 3:1,v:v); [α]^D^_25_ +48.18 (*c* 0.22, CH_3_OH); ^1^H NMR (400 MHz, CDCl_3_) δ 7.89 (d, *J* = 7.8 Hz, 1H), 7.49 (d, *J* = 7.2 Hz, 1H), 7.41 (t, *J* = 7.4 Hz, 1H), 7.26 (t, *J* = 7.6 Hz, 1H), 6.30 (d, *J* = 9.7 Hz, 2H), 5.92 (d, *J* = 3.7 Hz, 1H), 5.03 (t, *J* = 4.0 Hz, 1H), 4.94 (dd, *J* = 9.3, 4.8 Hz, 1H), 4.69 (dd, *J* = 12.2, 2.4 Hz, 1H), 4.56–4.47 (m, 1H), 4.40 (dd, *J* = 12.2, 5.4 Hz, 1H), 3.79 (s, 3H), 3.76 (s, 3H), 2.47 (t, *J* = 7.0 Hz, 2H), 2.35 (s, 3H), 1.63–1.57 (m, 2H), 1.55 (s, 3H), 1.47 (d, *J* = 7.8 Hz, 2H), 1.36 (s, 3H), 0.92 (t, *J* = 7.3 Hz, 3H); ^13^C NMR (101 MHz, CDCl_3_) δ 167.3, 166.0, 161.8, 158.8, 139.2, 134.4, 131.7, 131.3, 130.4, 127.1, 124.8, 115.1, 113.1, 106.8, 104.6, 96.4, 96.1, 79.1, 77.3, 75.6, 73.2, 63.4, 55.8, 55.4, 30.7, 26.6, 22.1, 20.1, 19.5, 13.7; HRMS (ESI) *m/z*: [M + Na]^+^ calcd for C_31_H_36_O_9_Na, 575.2257; found, 575.2293.

**Synthesis of 1,2-*O*-diacetyl-3-*O*-(2,4-dimethoxy-6-methylbenzoyl)-5-*O*-(2-(hex-1-yn-1-yl)benzoyl)-D-ribofuranose (16):** A solution of **15** (2.1 g, 3.8 mmol) in acetic acid (10 mL) and Ac_2_O (1.94 g, 19.0 mmol) was added concentrated sulfuric acid (0.2 mL) dropwise over 10 min. After addition, the reaction mixture was stirred at room temperature for 2 h. TLC detection showed the reaction was finished. The compound was diluted with CH_2_Cl_2_ (80 mL) and washed with water (100 mL × 3), sat*.* NaHCO_3_ (100 mL × 3), brine (100 mL), and dried (anhydrous Na_2_SO_4_). The obtained residue was purified by flash column chromatography to afford **16** as colourless oil (1.68 g, β:α 8:1, 74%). **16-**β**:** R*_f_* 0.30 (PE/EtOAc 4:1, v:v); [α]^D^_25_ −10.83 (*c* 0.23, CH_3_OH); ^1^H NMR (400 MHz, CDCl_3_) δ 7.95 (d, *J* = 7.9 Hz, 1H), 7.51 (d, *J* = 7.7 Hz, 1H), 7.42 (t, *J* = 7.6 Hz, 1H), 7.29 (t, *J* = 7.7 Hz, 1H), 6.31 (d, *J* = 2.0 Hz, 1H), 6.29 (d, *J* = 2.2 Hz, 1H), 6.19 (s, 1H), 5.64 (dd, *J* = 7.3, 4.9 Hz, 1H), 5.57 (d, *J* = 4.9 Hz, 1H), 4.72 (dd, *J* = 12.1, 3.1 Hz, 1H), 4.64–4.57 (m, 1H), 4.41 (dd, *J* = 12.2, 4.9 Hz, 1H), 3.80 (s, 3H), 3.76 (s, 3H), 2.47 (t, *J* = 7.1 Hz, 2H), 2.29 (s, 3H), 2.07 (s, 3H), 1.95 (s, 3H), 1.64–1.57 (m, 2H), 1.51–1.44 (m, 2H), 0.93 (t, *J* = 7.3 Hz, 3H); ^13^C NMR (101 MHz, CDCl_3_) δ 169.4, 169.2, 167.0, 165.8, 162.0, 158.9, 139.2, 134.5, 131.9, 131.4, 130.4, 127.2, 125.1, 114.7, 107.0, 98.4, 96.7, 96.3, 80.0, 79.1, 74.3, 71.1, 63.9, 55.9, 55.5, 30.8, 22.2, 21.0, 20.7, 20.1, 19.6, 13.8; HRMS (ESI) *m/z*: [M + Na]^+^ calcd for C_32_H_36_O_11_Na, 619.2150; found, 619.2147. **16-**α**:** R*_f_* 0.17 (PE/EtOAc 4:1, v:v); [α]^D^_25_ +10.20 (*c* 0.15, CH_3_OH); ^1^H NMR (400 MHz, CDCl_3_) δ 7.89 (dd, *J* = 7.9, 1.1 Hz, 1H), 7.52 (dd, *J* = 7.8, 1.0 Hz, 1H), 7.43 (td, *J* = 7.6, 1.4 Hz, 1H), 7.32 (td, *J* = 7.7, 1.3 Hz, 1H), 6.48 (d, *J* = 4.6 Hz, 1H), 6.33– 6.32 (m, 2H), 5.59 (dd, *J* = 6.8, 3.0 Hz, 1H), 5.40 (dd, *J* = 6.8, 4.6 Hz, 1H), 4.70–4.66 (m, 1H), 4.65 (dd, *J* = 12.1, 3.0 Hz, 1H), 4.54 (dd, *J* = 12.1, 3.7 Hz, 1H), 3.81 (s, 3H), 3.78 (s, 3H), 2.48 (t, *J* = 7.1 Hz, 2H), 2.36 (s, 3H), 2.06–2.05 (m, 6H), 1.63–1.60 (m, 2H), 1.54–1.43 (m, 2H), 0.94 (t, *J* = 7.3 Hz, 3H); ^13^C NMR (101 MHz, CDCl_3_) δ 169.8, 169.5, 167.4, 165.9, 161.9, 158.9, 138.7, 134.5, 131.9, 131.3, 130.2, 127.4, 124.9, 115.3, 106.9, 96.6, 96.4, 94.3, 82.2, 79.1, 70.6, 70.4, 64.2, 56.0, 55.5, 30.8, 22.2, 21.2, 20.5, 20.2, 19.6, 13.8; HRMS (EI) *m/z*: [M + Na]^+^ calcd for C_32_H_36_O_11_Na, 619.2150; found, 619.2150.

**Synthesis of 1-(2’-*O*-acetyl-3’-*O*-(2,4-dimethoxy-6-methylbenzoyl)-5’-*O*-(2-(hex-1-yn-1-yl)benzoyl)-β-D-ribofuranosyl)uracil (17):** To a suspension of uracil (0.24 g, 2.2 mmol) in dry MeCN (15 mL) was added BSA (1.36 g, 6.7 mmol). The mixture was heated at 50 °C for 20 min. After cooled to room temperature, a solution of **16** (1.00 g, 1.7 mmol) in dry MeCN (5 mL) along with TMSOTf (1.30 g, 5.9 mmol) were added to the above reaction mixture at 0 °C. The solution was stirred for 5 min before heating to 75 °C for 3–4 h. Then the reaction mixture was poured into cold sat. NaHCO_3_ solution (30 mL). It was extracted with CH_2_Cl_2_ (50 mL). The combined organic layer was washed with sat. aq NaHCO_3_ (100 mL × 2), brine (50 mL × 2), and dried with anhydrous Na_2_SO_4_. After filtration, the filtrate was evaporated under reduced pressure. The residue was purified by silica gel column chromatography (DCM/CH_3_OH, 10:1) to give nucleoside **17** as a white solid (0.96 g, 89%). R*_f_* 0.43 (CH_2_Cl_2_/CH_3_OH 30:1,v:v); mp 69–70 °C; [α]^D^_25_ −3.10 (*c* 0.28, CH_3_OH); ^1^H NMR (400 MHz, CDCl_3_) δ 9.53 (s, 1H), 7.88 (d, *J* = 7.4 Hz, 1H), 7.54 (d, *J* = 7.4 Hz, 1H), 7.46 (t, *J* = 7.6 Hz, 1H), 7.40–7.33 (m, 2H), 6.33 (s, 2H,), 6.19 (d, *J* = 6.1 Hz, 1H), 5.67–5.64 (m, 1H), 5.52 (d, *J* = 8.1 Hz, 1H), 5.42 (t, *J* = 5.9 Hz, 1H), 4.72 (dd, *J* = 12.4, 2.5 Hz, 1H), 4.64 (dd, *J* = 12.4, 3.1 Hz, 1H'), 4.58–4.55 (m, 1H), 3.81 (s, 3H), 3.80 (s, 3H), 2.46 (t, *J* = 7.0 Hz, 2H), 2.32 (s, 3H), 2.05 (s, 3H), 1.63–1.55 (m, 2H), 1.51–1.41 (m, 2H), 0.92 (t, *J* = 7.4 Hz, 3H); ^13^C NMR (101 MHz, CDCl_3_) δ 169.7, 166.9, 166.0, 163.1, 162.1, 159.1, 150.5, 139.3, 134.5, 132.2, 131.4, 129.8, 127.6, 124.6, 114.4, 107.1 103.4, 96.8, 96.4, 86.7, 80.6, 78.9, 73.0, 71.1, 63.8, 56.0, 55.5, 30.7, 22.1, 20.6, 20.2, 19.5, 13.7; HRMS (ESI) *m/z*: [M + Na]^+^ calcd for C_34_H_36_N_2_O_11_Na, 671.2217; found, 671.2214.

**Synthesis of kipukasin A:** To a solution of nucleoside **17** (0.60 g, 0.92 mmol) in dry CH_2_Cl_2_ (15 mL) was added H_2_O (1.0 equiv), and ethanol (6.0 equiv) under an argon atmosphere. The mixture was stirred at room temperature for 20 minutes. A freshly prepared solution of Ph_3_PAuOTf in CH_2_Cl_2_ (5 mol % in 1.0 mL) was added, and stirring was continued at room temperature for 5 hours until nucleoside **17** was consumed as monitored by TLC. The reaction mixture was filtered with celite. After filtration, the filtrate was evaporated to dryness under reduced pressure. The obtained residue was recrystallized in petroleum ether (5 mL) to provide kipukasin A as a white powder solid (387 mg, 90%). R*_f_* 0.36 (CH_2_Cl_2_/CH_3_OH 25:1,v:v); mp 95–96 °C; [α]^D^_25_ −37.50 (*c* 0.12, CH_3_OH) (lit. [11] [α]^D^_25_ −26 (*c* 0.12, CH_3_OH)); ^1^H NMR (400 MHz, CDCl_3_) δ 9.32 (s, 1H, NH), 7.81 (d, *J* = 8.2 Hz, 1H, H-6), 6.33 (s, 2H, H-3'', H-5''), 6.14 (d, *J* = 6.7 Hz, 1H, H-1'), 5.79 (d, *J* = 8.1 Hz, 1H, H-5), 5.68 (dd, *J* = 6.4, 2.8 Hz, 1H, H-3'), 5.56 (dd, *J* = 6.6, 5.7 Hz, 1H, H-2'), 4.33–4.32 (m, 1H, H-4'), 3.96 (s, 2H, H-5'), 3.81 (s, 6H, OMe-2'', OMe-4''), 3.32 (s, 1H, -OH), 2.32 (s, 3H, Me-6''), 2.06 (s, 3H, Me-6'); ^13^C NMR (101 MHz, CDCl_3_) δ 170.1 (C-6'), 167.3 (C-7''), 163.4 (C-4), 162.1 (C-2''), 159.1 (C-4''), 150.7 (C-2), 140.9 (C-6), 139.2 (C-6''), 114.7 (C-1''), 107.1 (C-5''), 103.4 (C-5), 96.4 (C-3''), 87.4 (C-1'), 84.0 (C-4'), 73.2 (C-2'), 72.1 (C-3'), 62.2 (C-5'), 56.0 (OMe-4''), 55.5 (OMe-2''), 20.7 (Me-6'), 20.3 (Me-6''); HRMS (ESI) *m/z*: [M + Na]^+^ calcd for C_21_H_24_N_2_O_10_Na, 487.1329; found, 487.1327.

## Supporting Information

File 1 Experimental procedures of compounds **6**–**9**, copies of ^1^H and ^13^C NMR spectra of all compounds and X-ray crystal data of compound **13**.
