# Supplementary material for: First total synthesis of kipukasin A
Source: Beilstein J Org Chem. 2017 May 9;13:855–62. doi: 10.3762/bjoc.13.86 (PMC5433220; doi:10.3762/bjoc.13.86)
Supplement: File 1 — Experimental procedures of compounds 6–9, copies of 1H and 13C NMR spectra of all compounds and X-ray crystal data of compound 13. [file Beilstein_J_Org_Chem-13-855-s001.pdf]

Supporting Information

for

First total synthesis of kipukasin A

Chuang Li, Haixin Ding*, Zhizhong Ruan, Yirong Zhou and Qiang Xiao*

Address: Jiangxi Key Laboratory of Organic Chemistry, Jiangxi Science & Technology Normal University, Nanchang, Jiangxi 330013, China

Email: Haixin Ding* - dinghaixin_2010@163.com; Qiang Xiao* - xiaoqiang@tsinghua.org.cn

* Corresponding author

Experimental procedures of compounds 6–9, copies of ^{1}H and ^{13}C NMR spectra of all compounds and X-ray crystal data of compound 13

Table of contents

Experimental section……………………………………………………………………S2

Copies of ^{1}H NMR, ^{13}C NMR pectra of all compounds…………………………………S5

X-ray crystal structure of compound **13**………………………………………………S16

Experimental section

Synthesis of 2,4-dihydroxy-6-methylbenzaldehyde (6)

To a stirred solution of dry DMF (30 mL) was slowly added $POCl_3$ (12.01 mL, 128.9 mmol) dropwise at 0 °C under argon. The reaction mixture was stirred for 0.5 h, then the 1,3-dihydroxy-5-methylbenzene (**5**, 8.00 g, 64.4 mmol, dissolved in 30 mL of dry DMF) was slowly added into the mixture. The reaction mixture was stirred overnight and quenched with ice water. Then, an aqueous NaOH solution (10%) was added to the mixture until the pH of the solution was 10. The solution was heated to reflux for 10 min. After cooling, the mixture was stirred at 0 °C and concentrated hydrochloric acid was added until the pH of solution was 3, then precipitated to afford white powder solid **6** (7.40 g, 75%). R_f = 0.65 (PE: EtOAc = 2:1, v:v); mp 182-183 °C; ^{1}H NMR (400 MHz, DMSO-d_6) δ 12.07 (s, 1H), 10.69 (s, 1H), 10.04 (s, 1H), 6.20 (d, J = 1.4 Hz, 1H), 6.12 (d, J = 2.2 Hz, 1H), 2.44 (s, 3H, Me); ^{13}C NMR (101 MHz, DMSO-d_6) δ 193.0, 165.4, 165.3, 144.8, 112.7, 110.8, 100.3, 18.6.

Synthesis of 2,4-dimethoxy-6-methylbenzaldehyde (7)

To a solution of **6** (6.40 g, 42.1 mmol) in dry acetone (100 mL) was added K_2CO_3 (37.79 g, 273.4 mmol) and MeI (30.13 mL, 483.7 mmol) under argon. After addition, the reaction mixture was stirred for 6 h at room temperature. The reaction mixture was filtered with celite, and evaporated under reduced pressure. The residue was dissolved with diethyl ether (340 mL) and washed with water (2 × 100 mL), brine (2 × 50 mL), and dried (anhydrous Na_2SO_4). After filtration, the filtrated was evaporated to dryness under reduced pressure and gave the yellow powder solid **7** (7.03 g, 93%). R_f = 0.50 (PE: EtOAc = 4 :1, v:v); mp 64-65 °C; ^{1}H NMR (400 MHz, $CDCl_3$) δ 10.46 (s,

1H), 6.29 (s, 2H), 3.85 (s, 3H), 3.83 (s, 3H), 2.56 (s, 3H); ^{13}C NMR (101 MHz, $CDCl_3$) *δ* 190.6, 165.2, 164.5, 144.7, 117.4, 108.8, 95.8, 55.8, 55.5, 22.4.

Synthesis of 2,4-dimethoxy-6-methylbenzoic acid (8)

To the solution of **7** (6.00 g, 33.3 mmol) in the 100 mL of DMSO was added an aqueous NaH_2PO_4 solution (2.00 g, 16.7 mmol, dissolved in 15 mL of water). Then, the mixture was added aqueous $NaClO_2$ solution (purity: 80%, 5.50 g, 48.6 mmol, dissolved in 50 mL of water) dropwise at 0 °C. After addition, the mixture was stirred overnight at room temperature. The mixture was added the sat. $NaHCO_3$ at 0 °C until the pH of the solution was 9, the color of the solution turned to green. The solution was extracted with EtOAc (50 mL). Then the water layer was added the concentrated hydrochloric acid until the pH of the solution was 3. The mixture was stirred at 0 °C for 1 h while a solid precipitated. After filtration the solid was washed adequately and dried to obtain a white powder solid (4.5 g). The filtrate was extracted with EtOAc (50 mL × 2), and the organic layer was evaporated to dryness under reduced pressure. The residue was added 0.5 N hydrochloric acid (20 mL) while a solid precipitated. The solid was filtered washed adequately and dried to get white powder solid (0.8 g), the product **8** (5.3 g, 81%) was obtained. R_f = 0.25 (CH_2Cl_2:CH_3OH = 10:1, v:v); m.p.138-139 °C; ^{1}H NMR (400 MHz, DMSO-d_6) δ 6.43 (s, 1H), 6.40 (s, 1H), 3.76 (s, 3H), 3.74 (s, 3H), 2.21 (s, 3H); ^{13}C NMR (101 MHz, DMSO-d_6) δ 168.8, 160.5, 157.2, 136.42, 117.8, 106.6, 96.1, 55.7, 55.3, 19.4.

Synthesis of 2,4-dimethoxy-6-methylbenzoyl chloride (9)

The 2,4-dimethoxy-6-methylbenzoic acid (**8**, 4.0 g, 20.4 mmol) was dissolved in dry CH_2Cl_2 (25 mL) and $(COCl)_2$ (5.18 g, 40.8 mmol) was slowly added at 0 °C. After addition, the mixture was stirred overnight at room temperautre. The solvent was

evaporated under reduced pressure. The residue applied directly to the next reaction (R_f = 0.35, PE: EtOAc = 3:1, v:v).

Copies of ^{1}H NMR, ^{13}C NMR spectra of all compounds

^{1}H NMR Spectrum of compound **6**

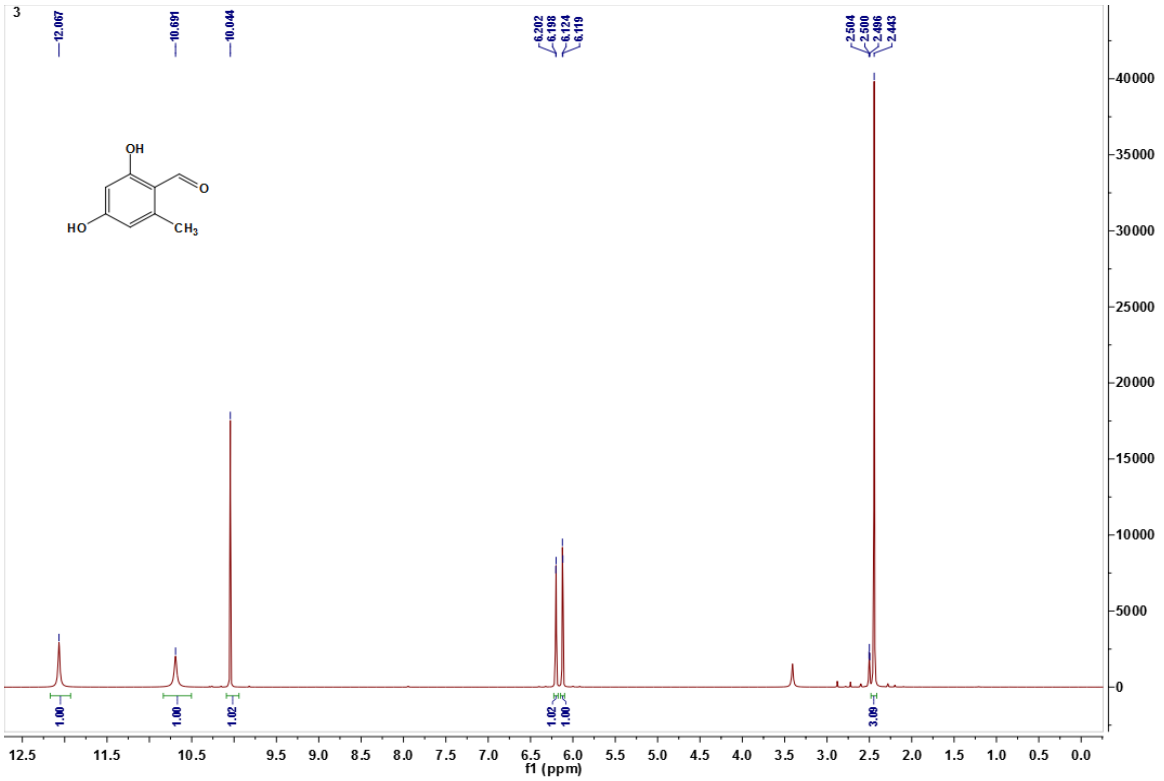

^{13}C NMR Spectrum of compound **6**

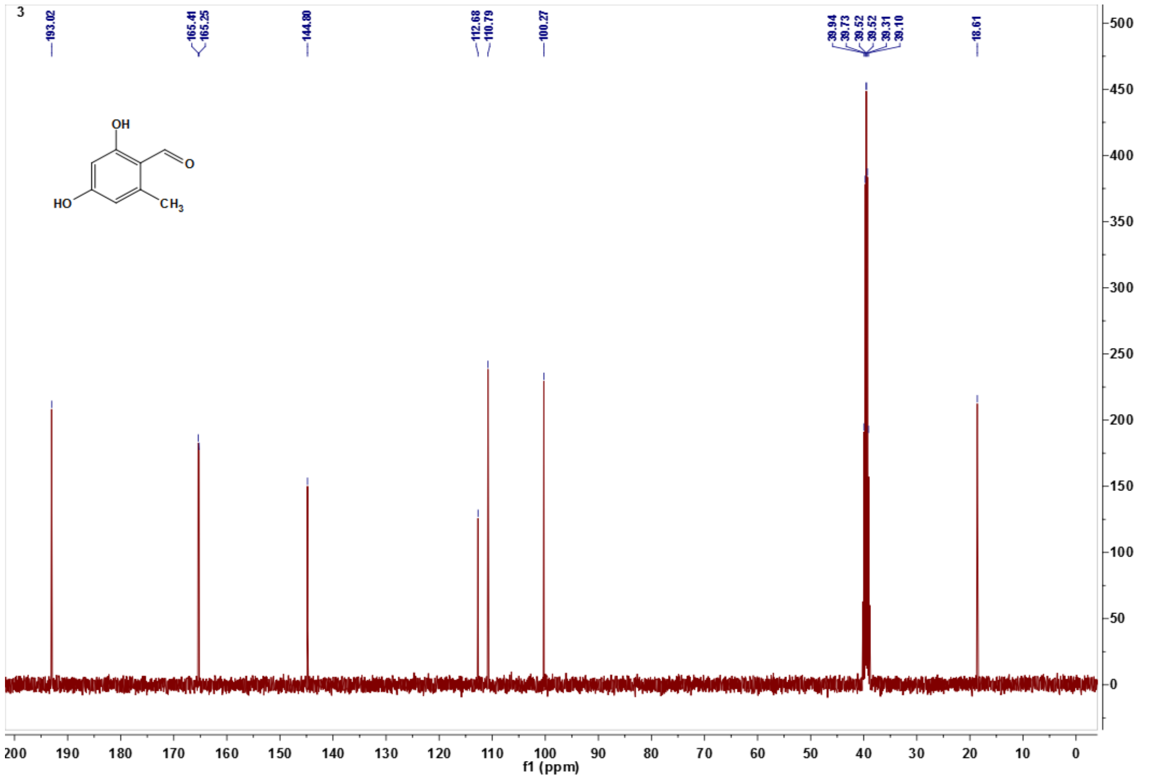

^{1}H NMR Spectrum of compound **7**

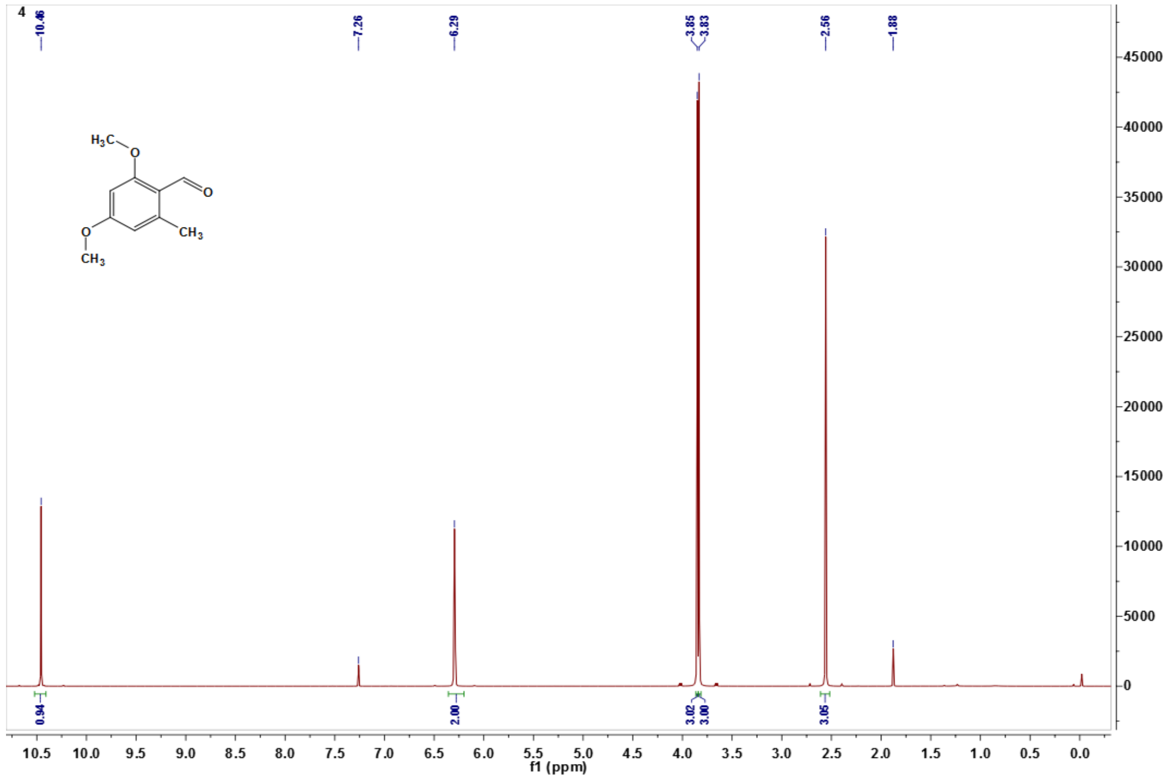

^{13}C NMR Spectrum of compound **7**

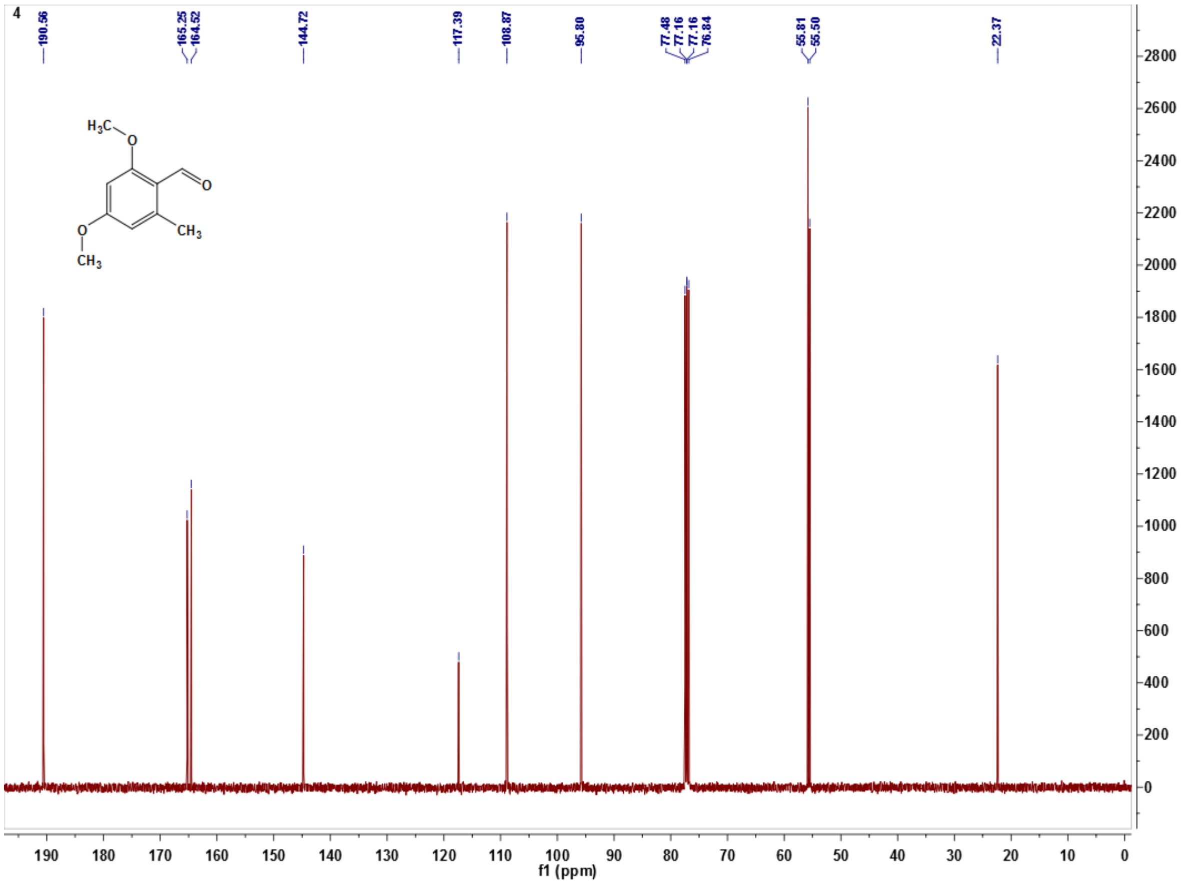

^{1}H NMR Spectrum of compound **8**

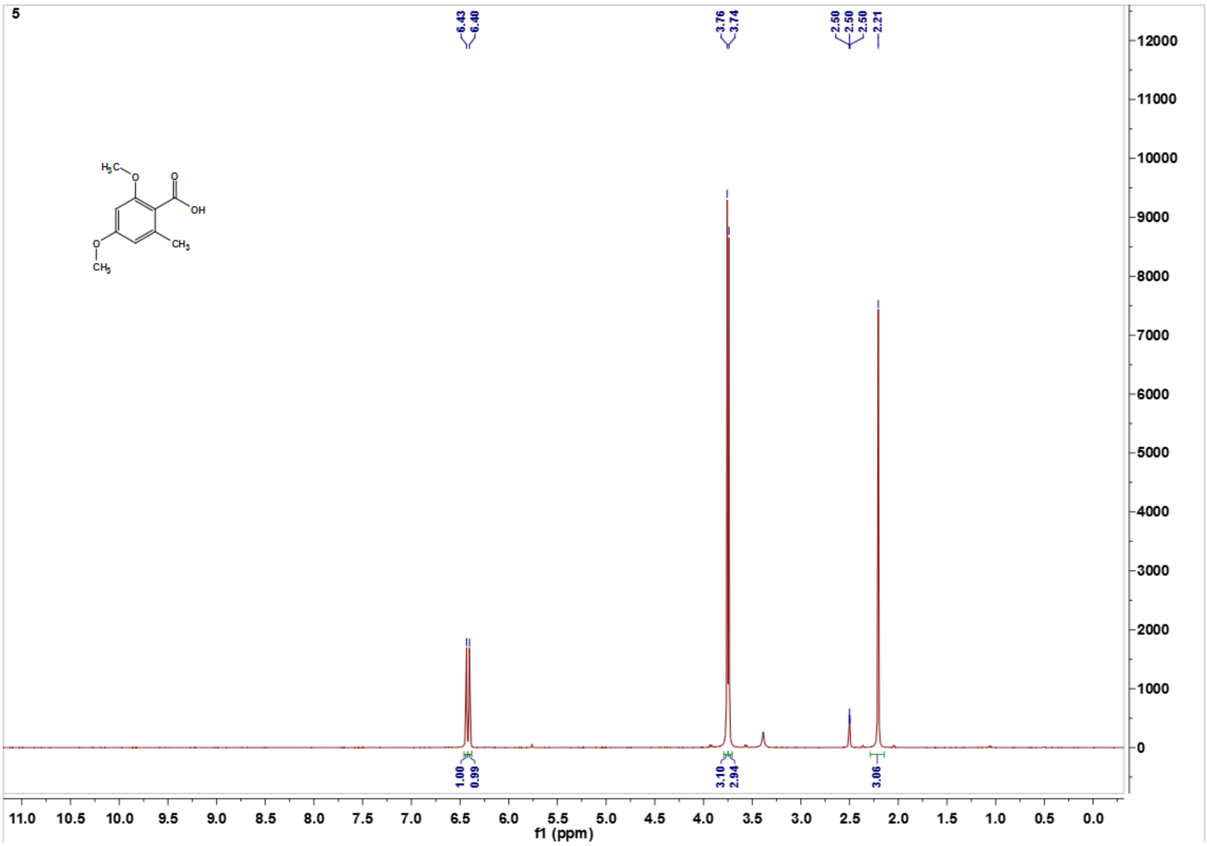

^{13}C NMR Spectrum of compound **8**

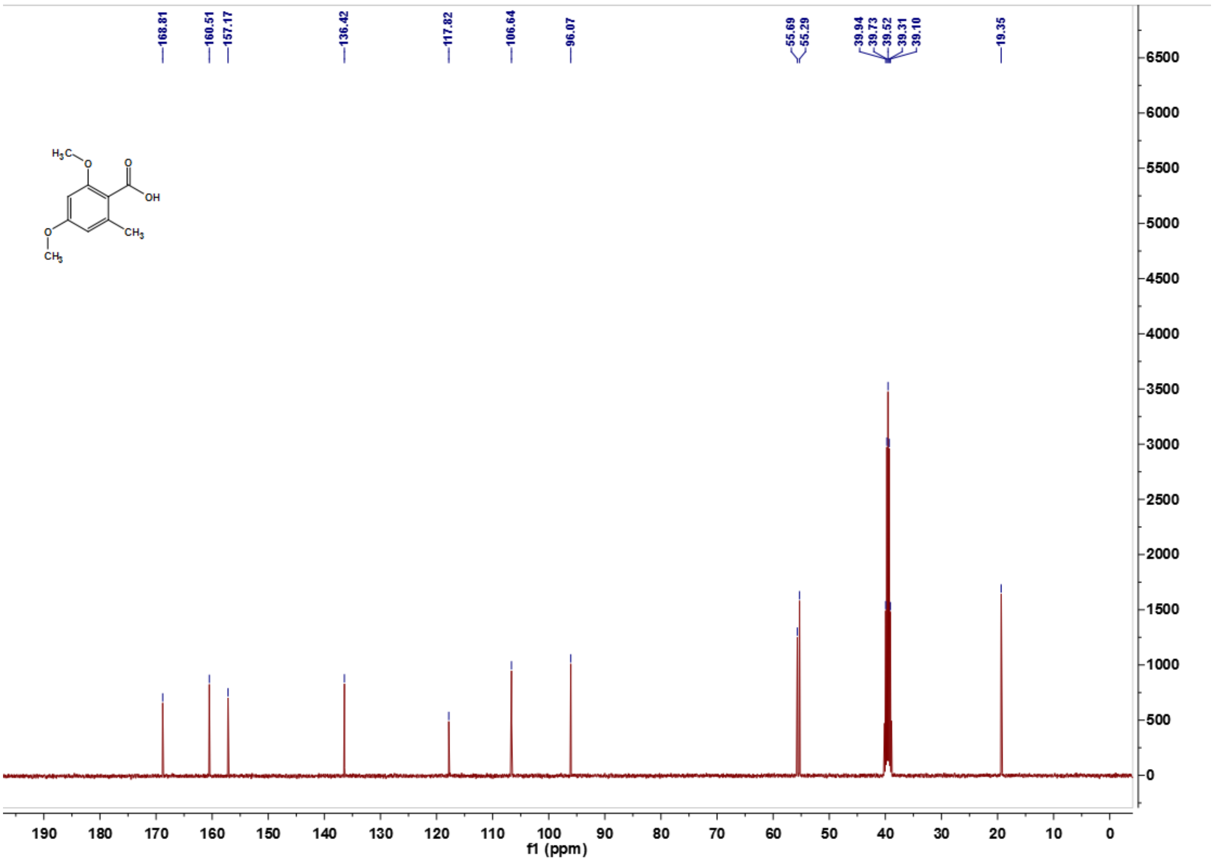

^{1}H NMR Spectrum of compound **11**

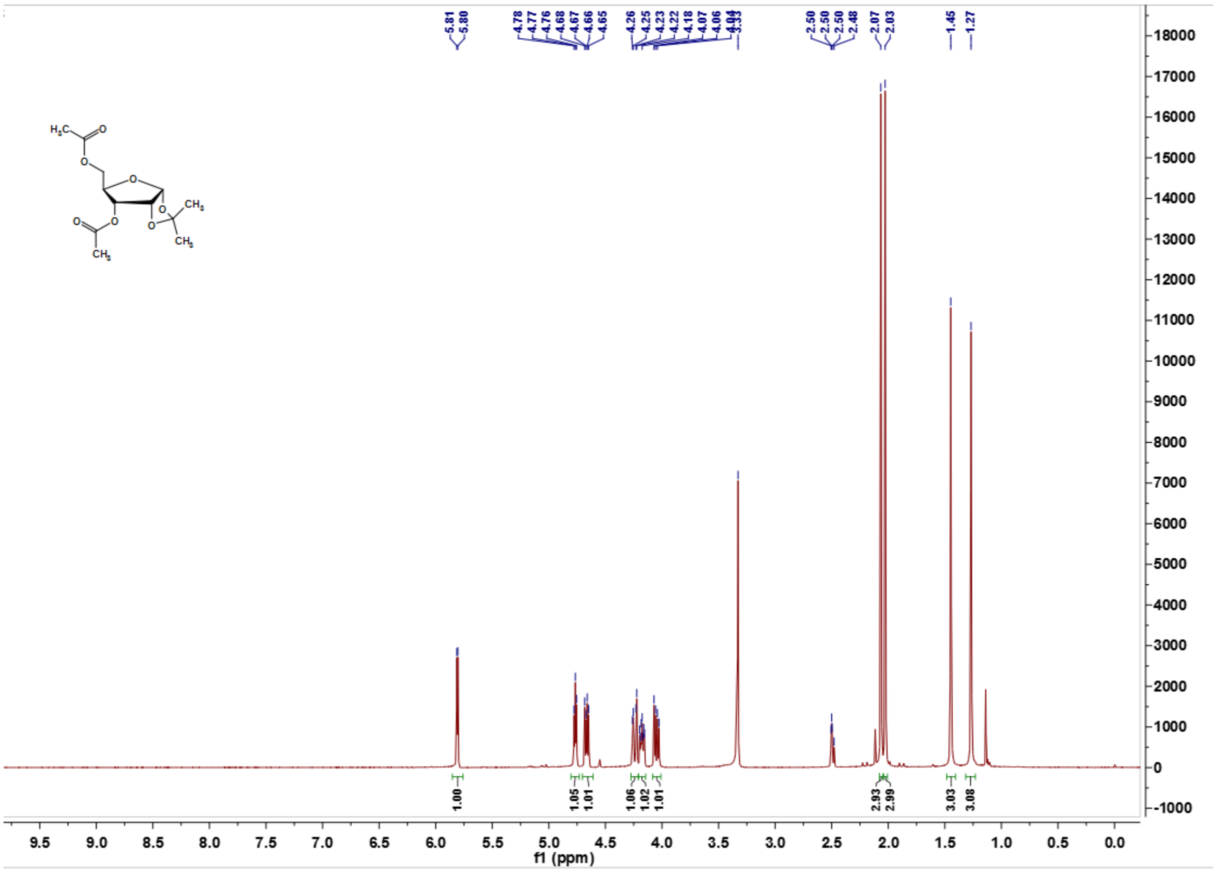

^{13}C NMR Spectrum of compound **11**

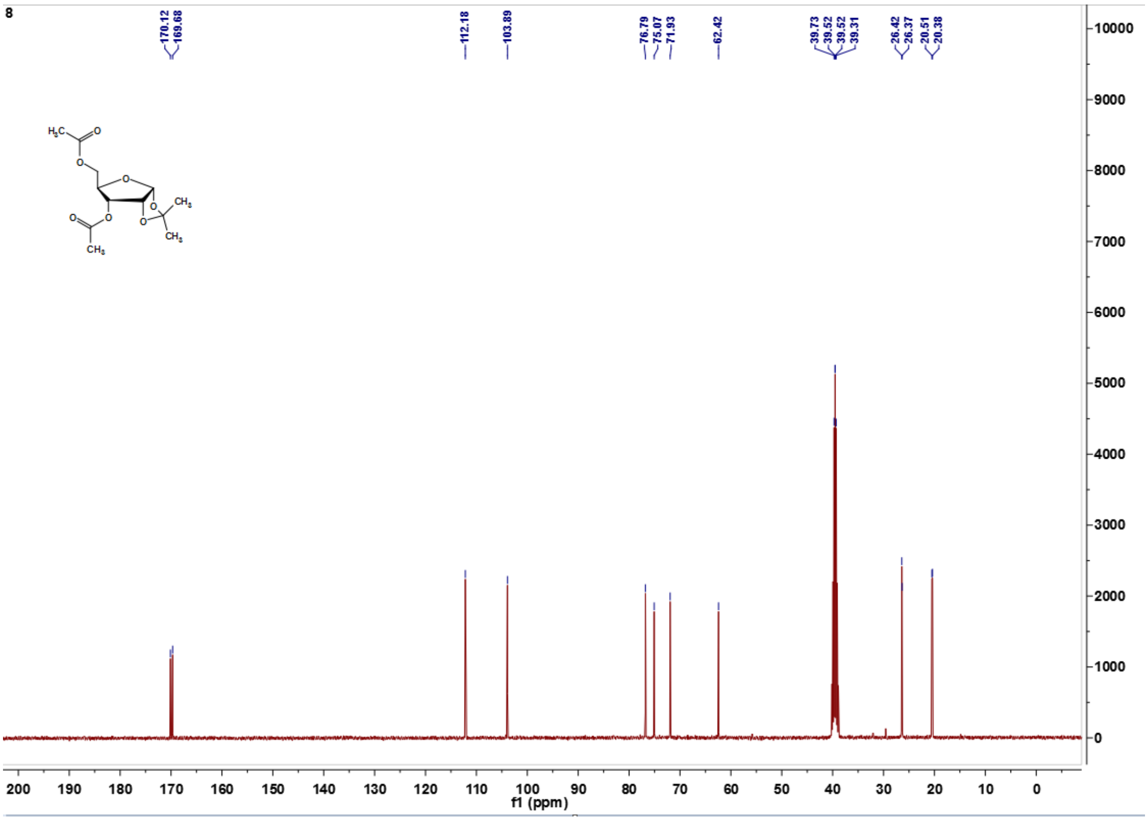

^{1}H NMR Spectrum of compound **12**

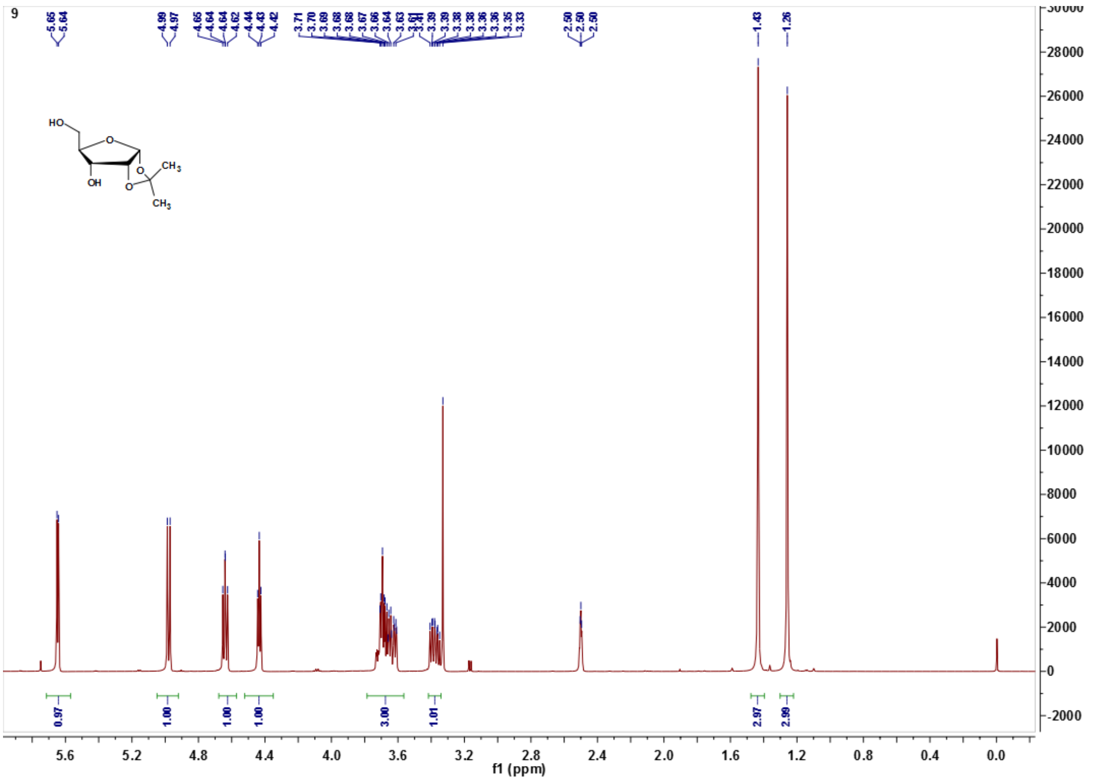

^{13}C NMR Spectrum of compound **12**

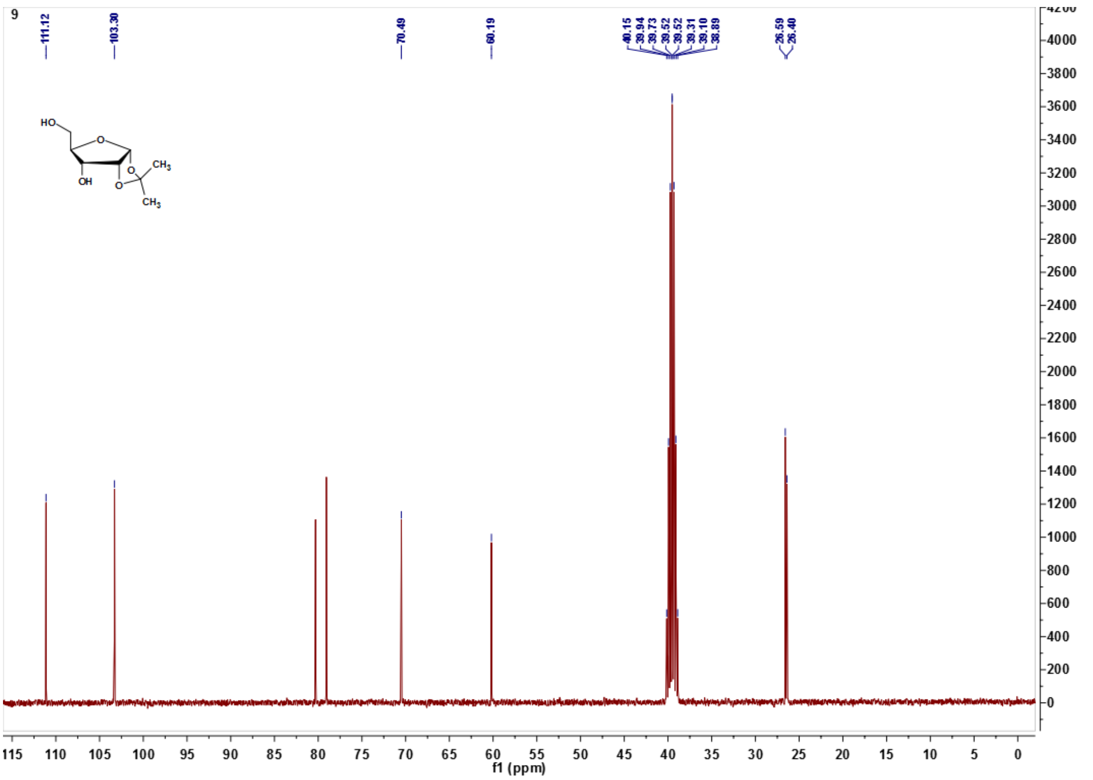

^{1}H NMR Spectrum of compound **13**

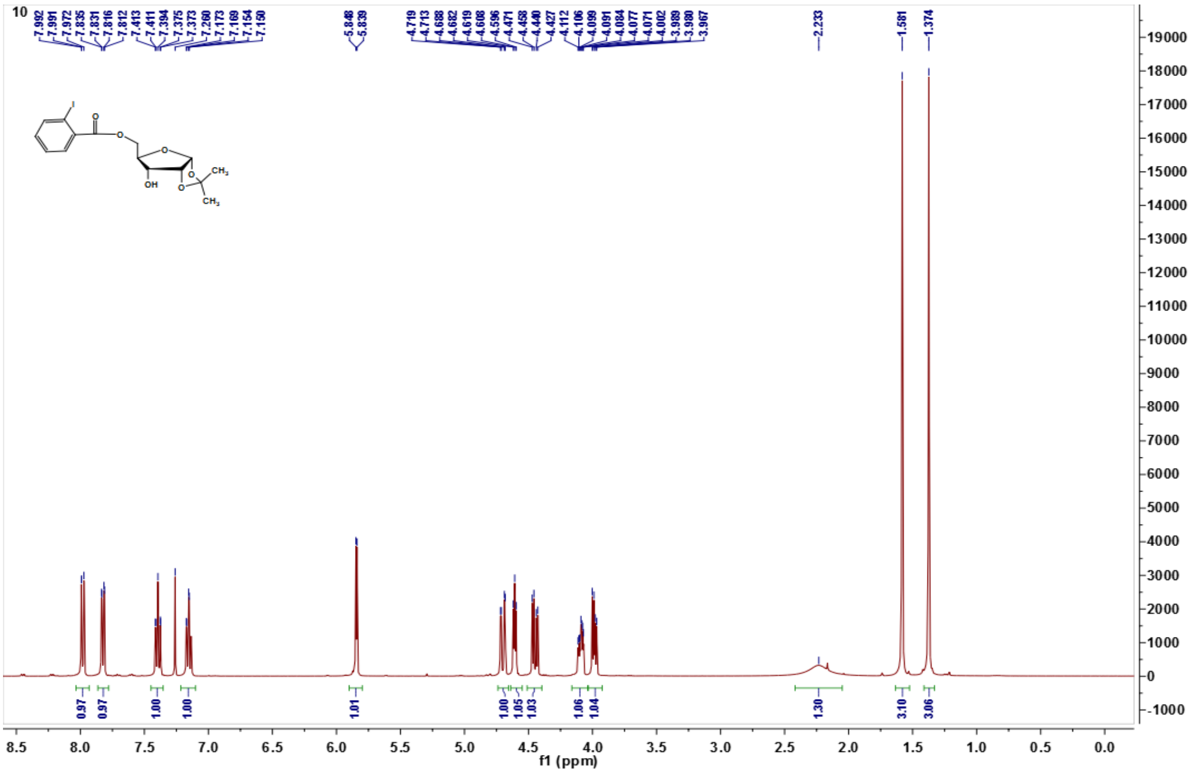

^{13}C NMR Spectrum of compound **13**

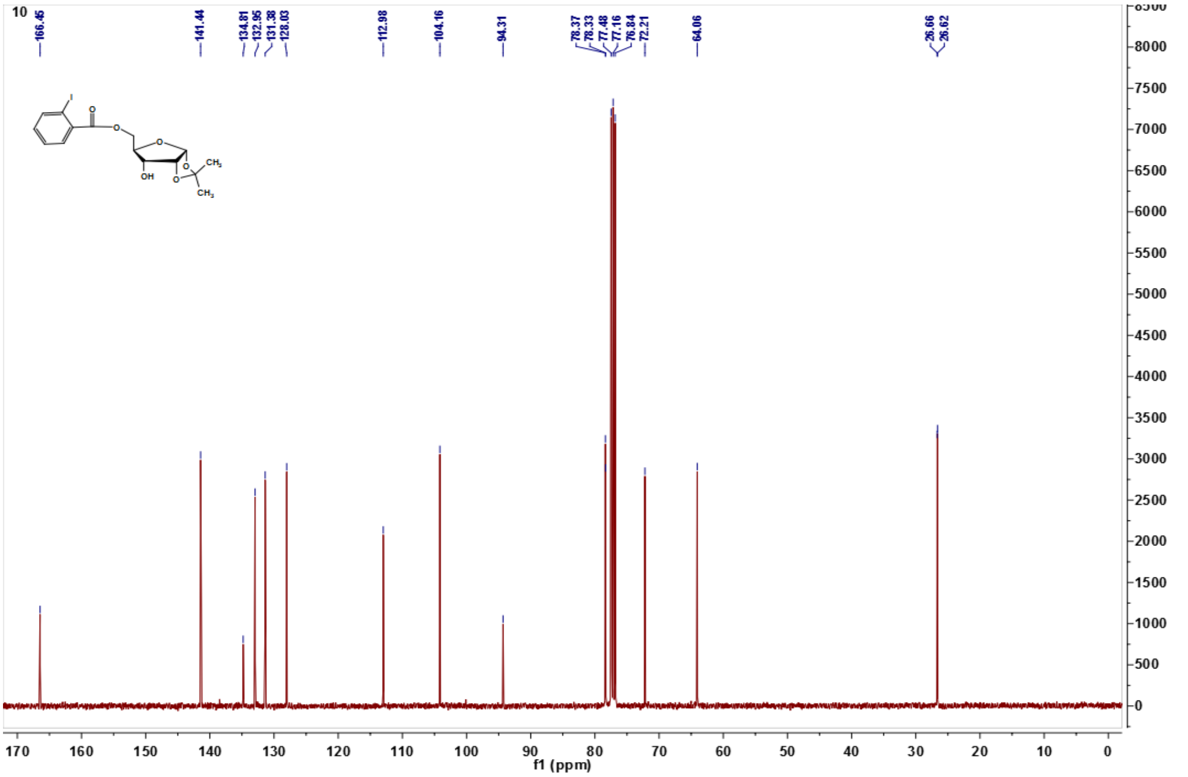

^{1}H NMR Spectrum of compound **14**

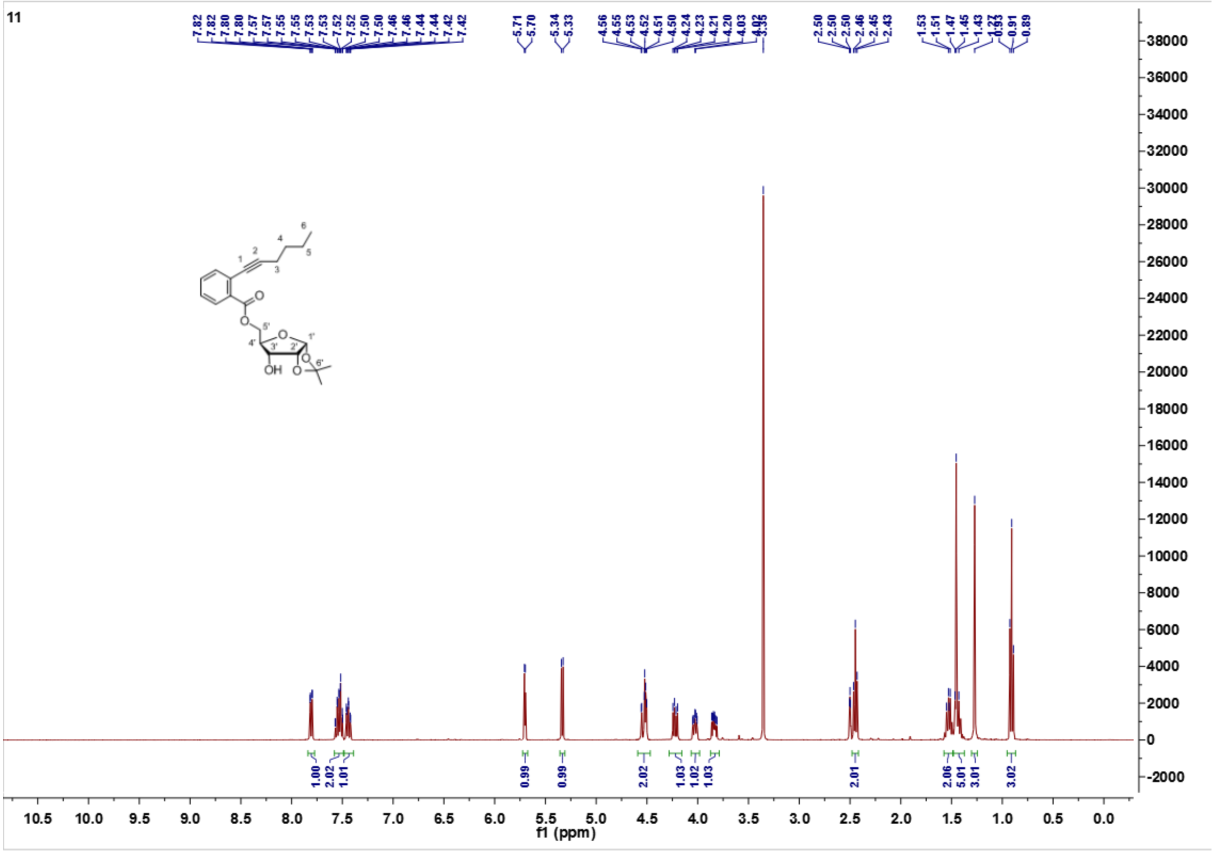

^{13}C NMR Spectrum of compound **14**

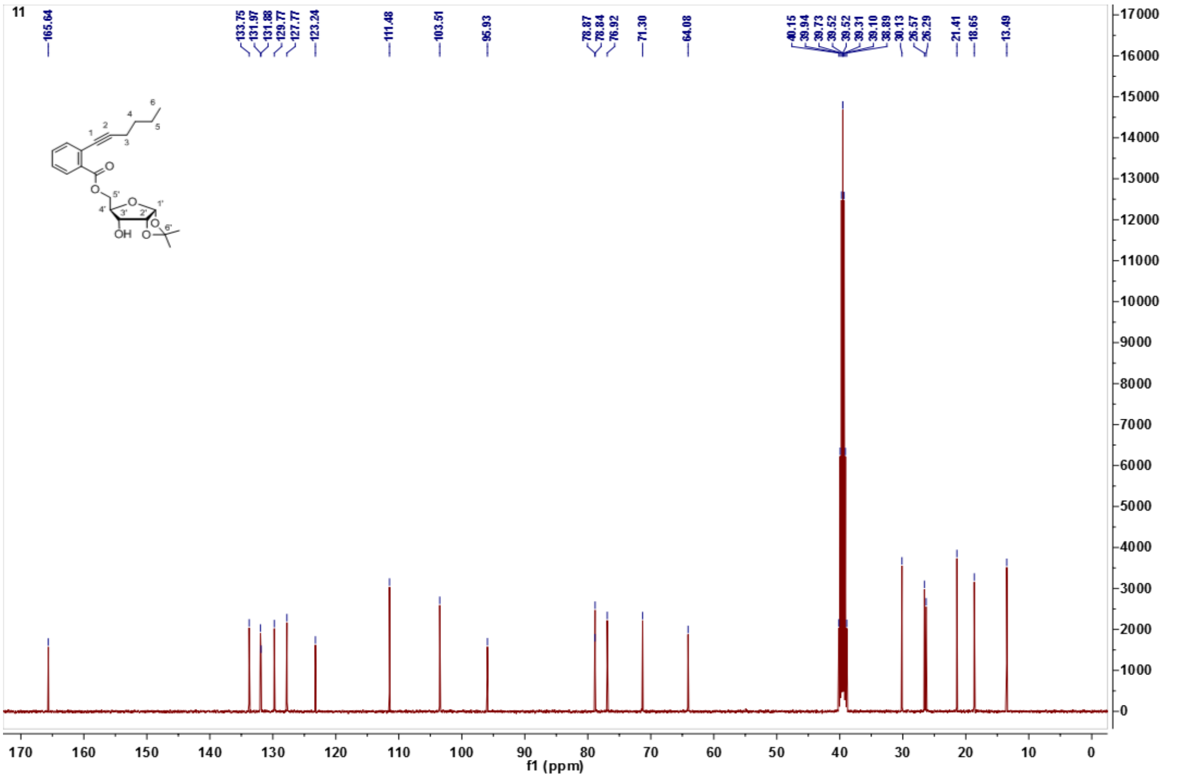

^{1}H NMR Spectrum of compound **15**

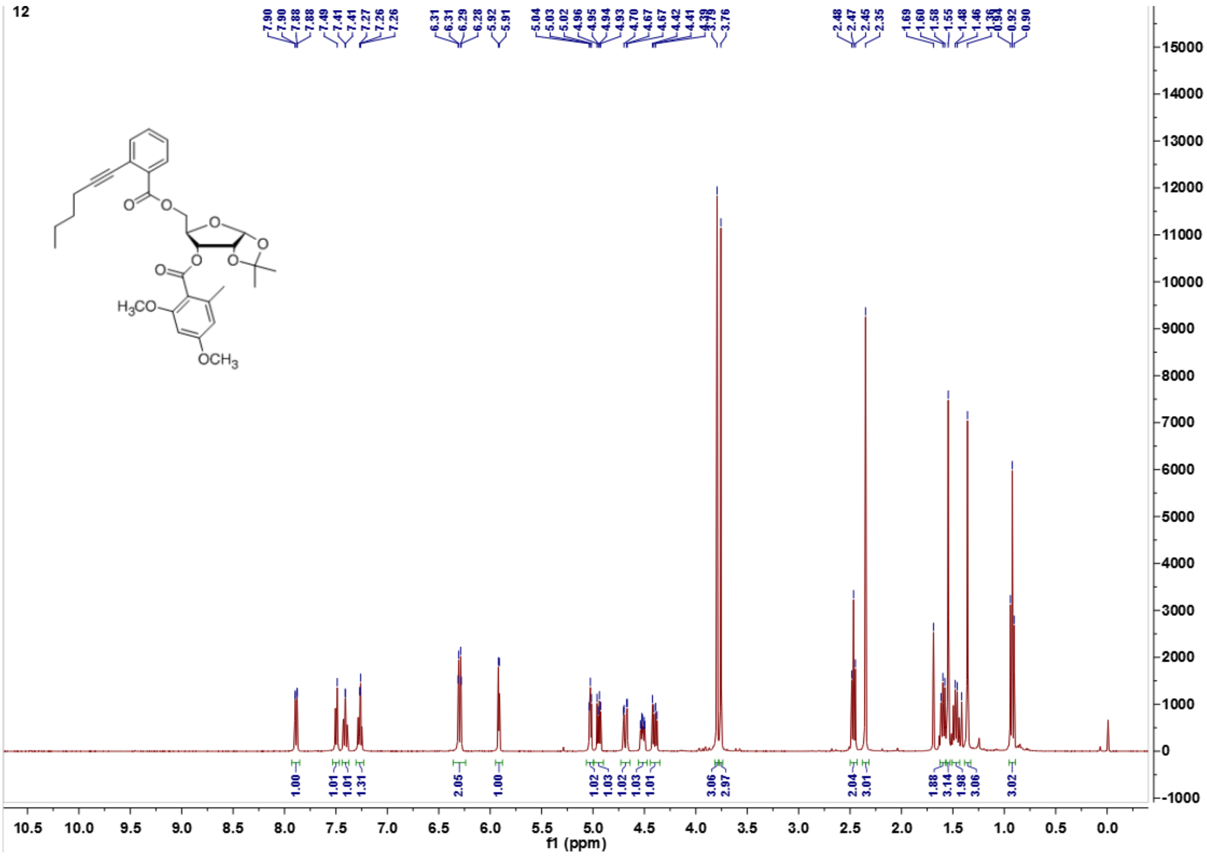

^{13}C NMR Spectrum of compound **15**

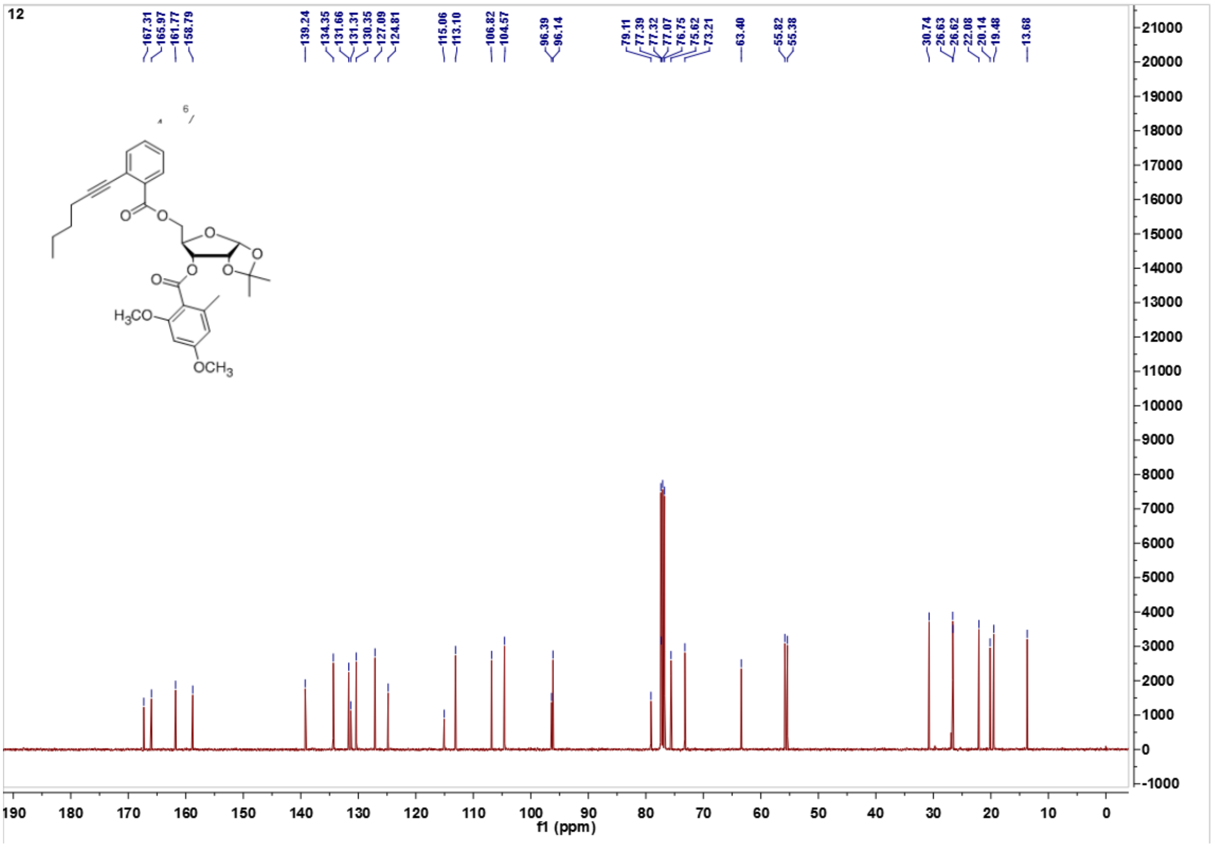

^{1}H NMR Spectrum of compound **16-** β

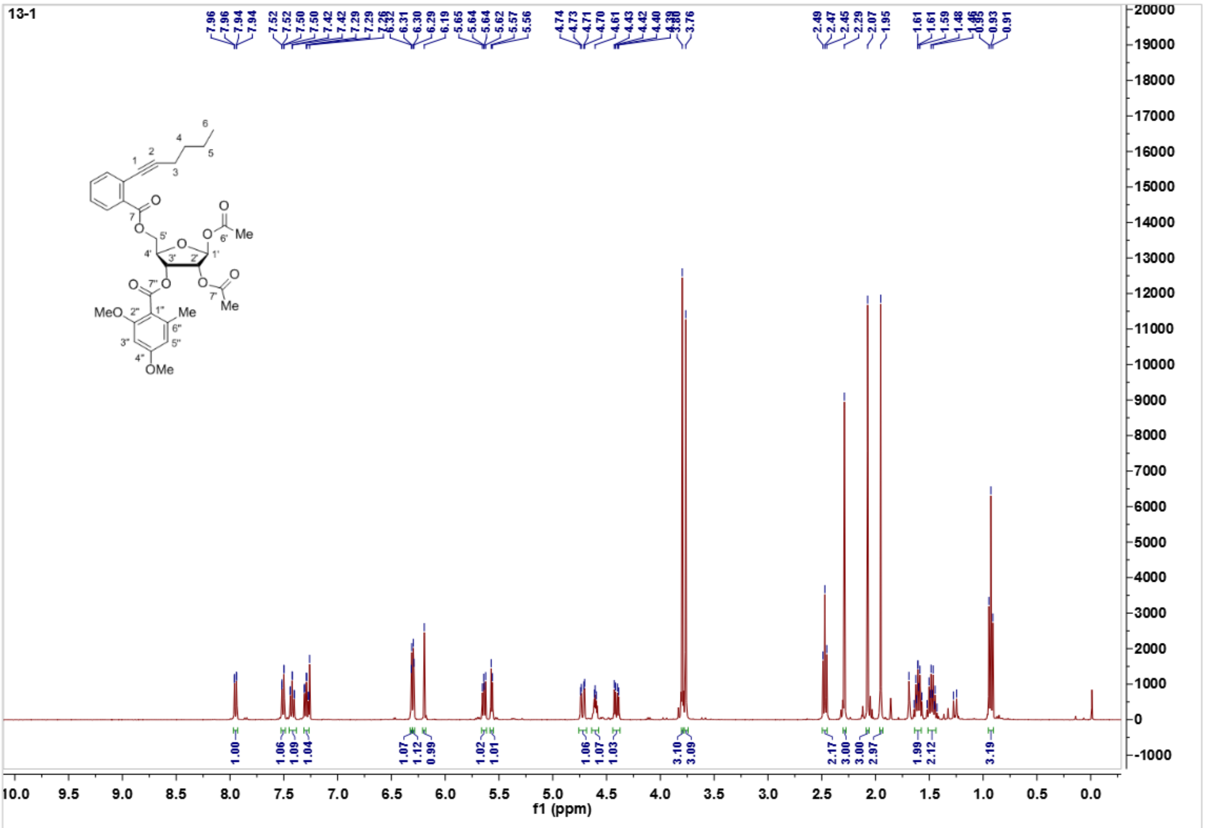

^{13}C NMR Spectrum of compound **16-** β

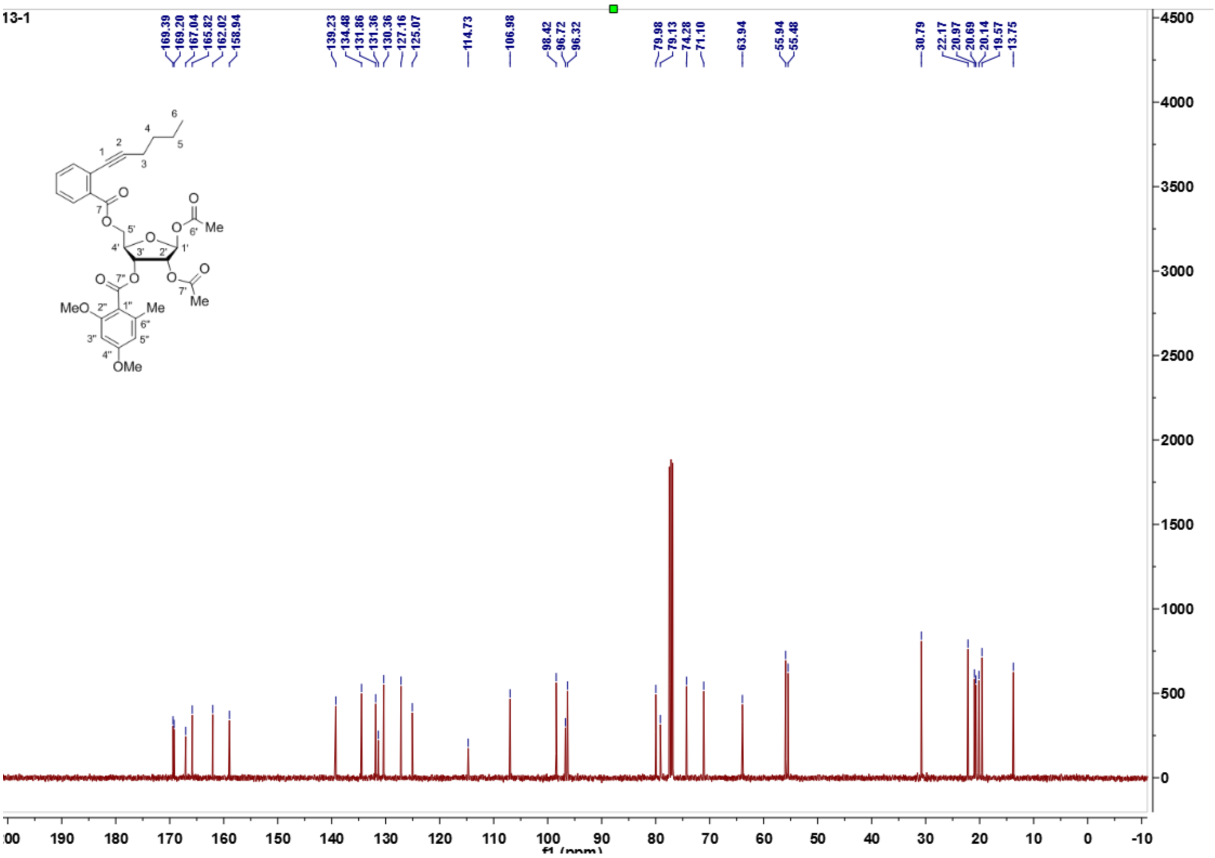

^{1}H NMR Spectrum of compound **16-** α

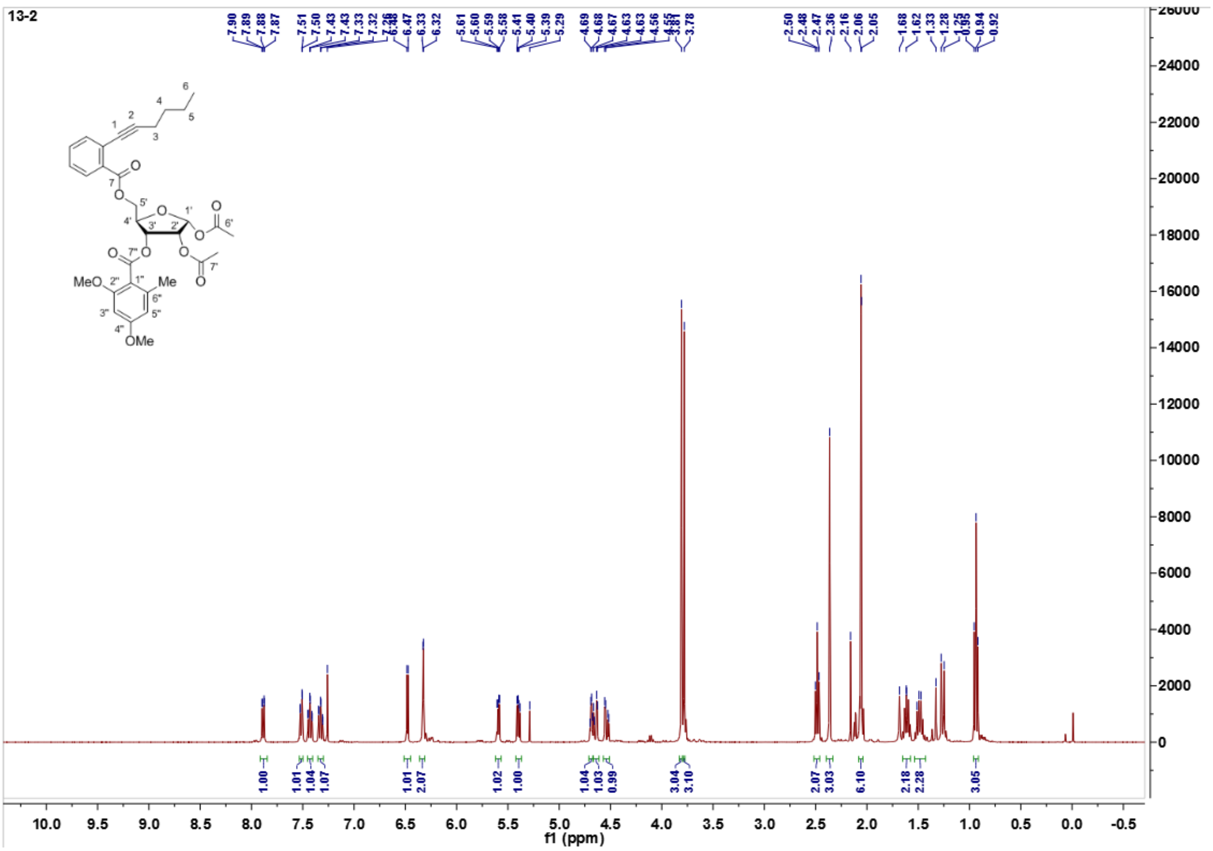

^{13}C NMR Spectrum of compound **16-** α

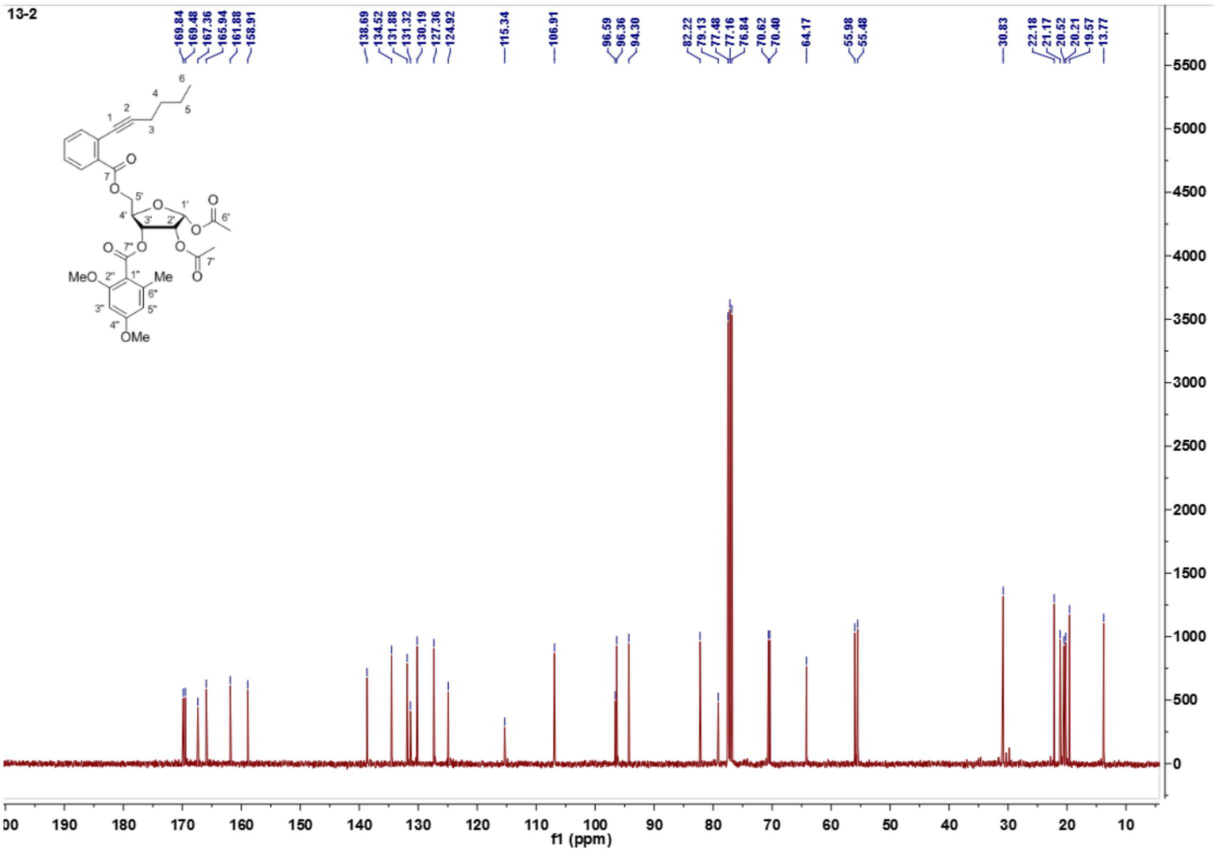

^{1}H NMR Spectrum of compound **17**

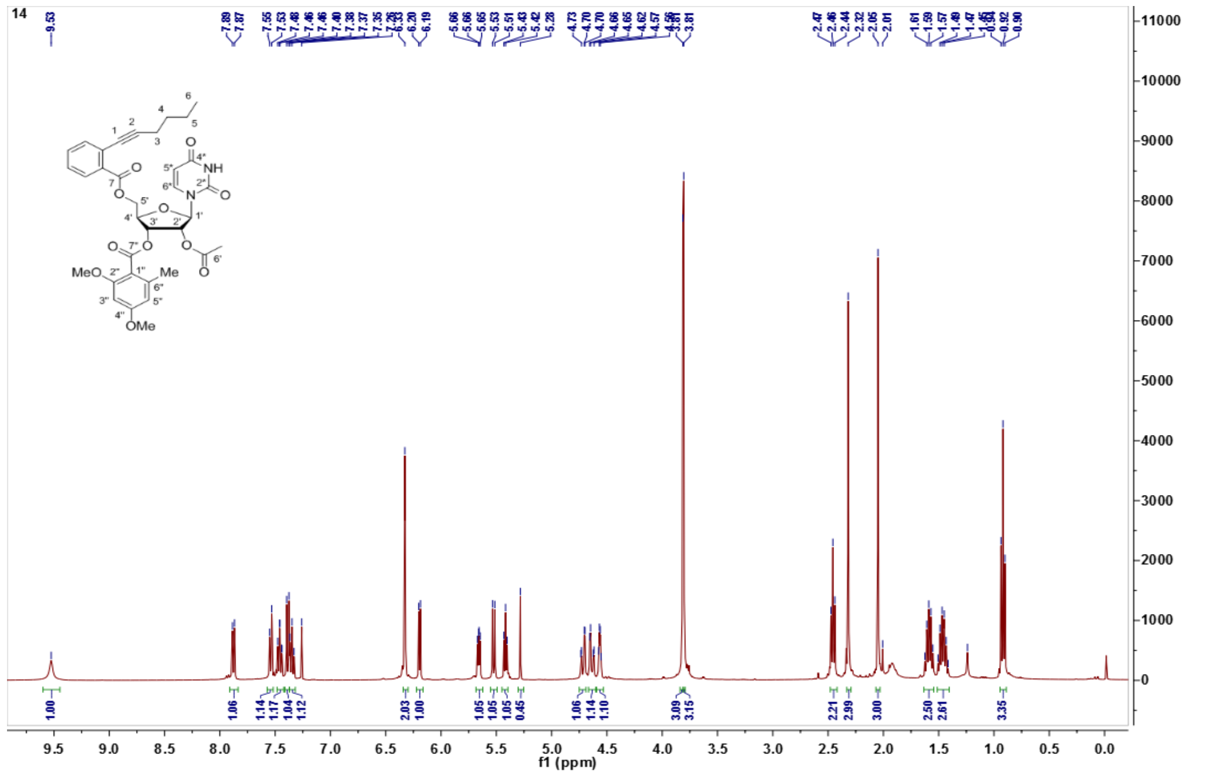

^{13}C NMR Spectrum of compound **17**

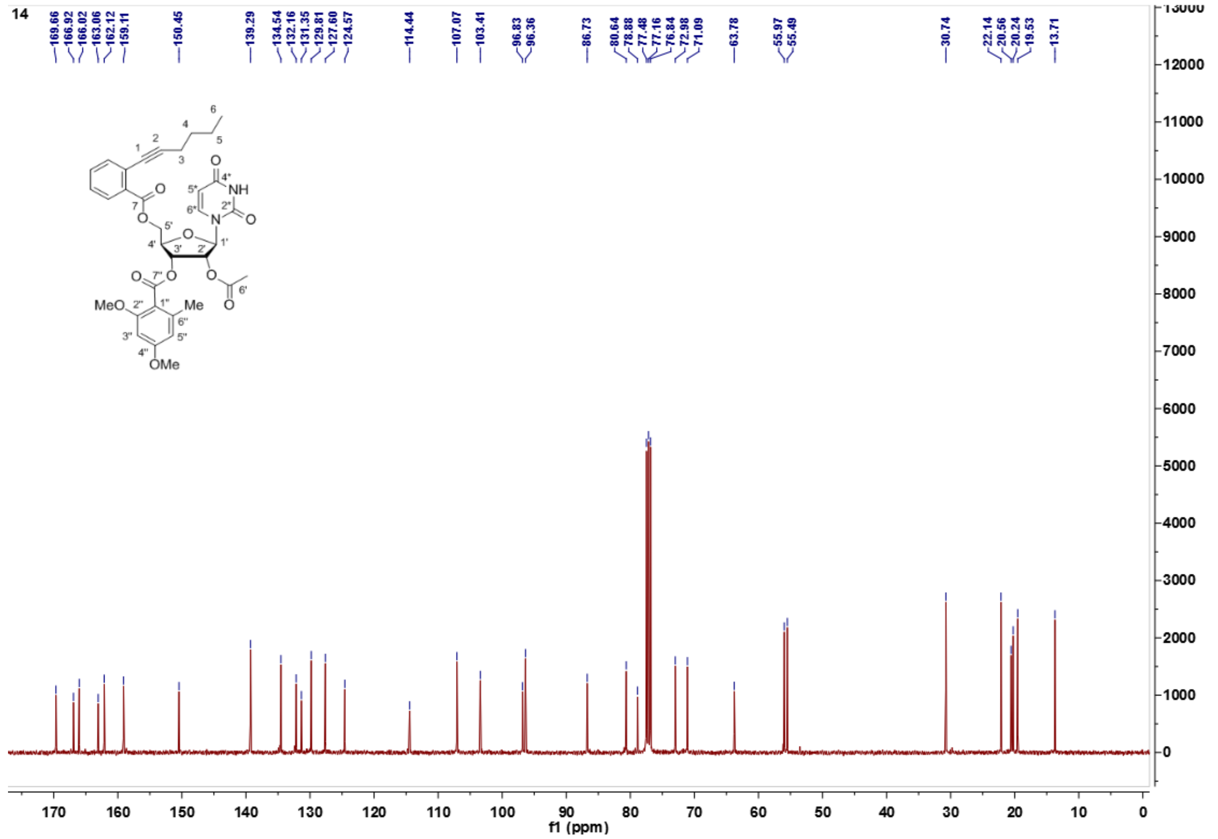

^{1}H NMR Spectrum of compound **Kipukasin A**

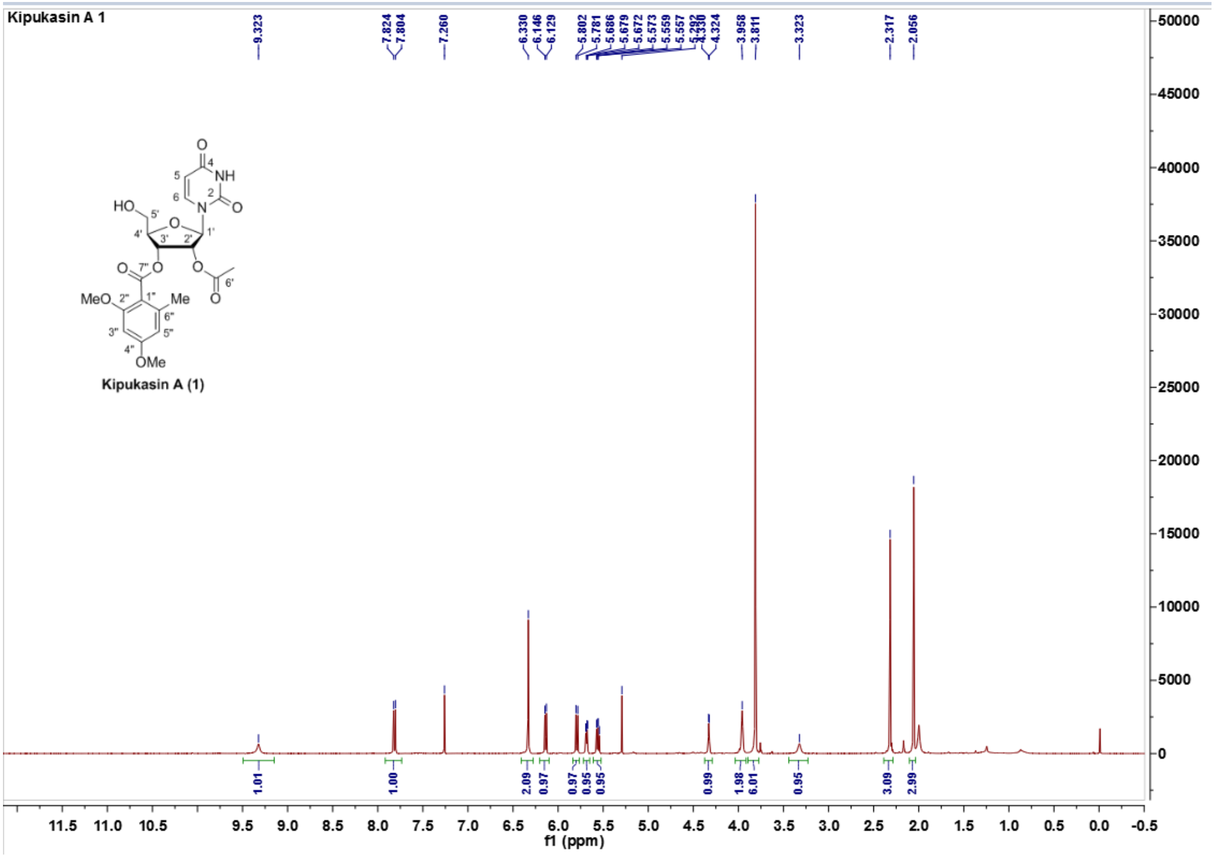

^{13}C NMR Spectrum of compound **Kipukasin A**

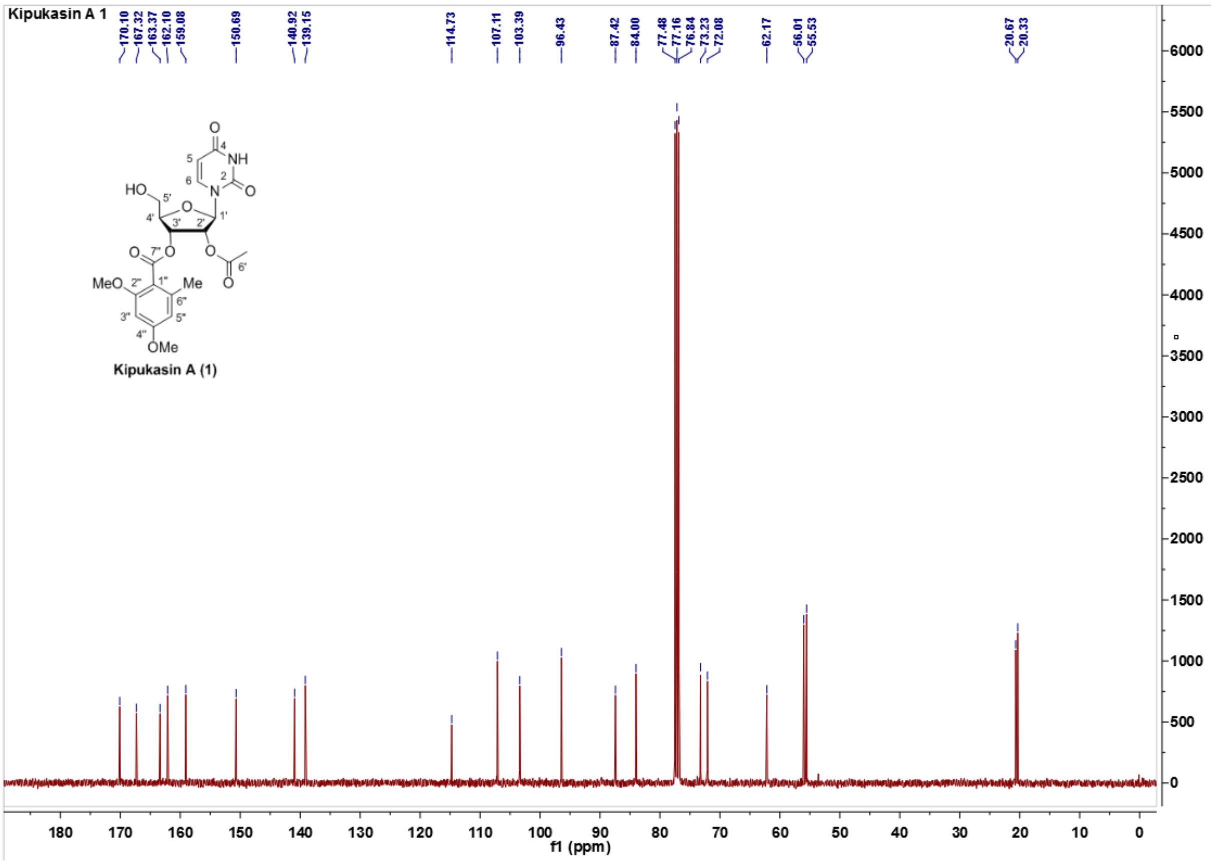

Table S1: Details of Data Collection, Processing and Structure Refinement

Sample code	**compound 13**		
Molecular formula	$C_{15}H_{17}IO_6$		
Molecular weight	420.18		
Color and habit	colorless block		
Crystal size	0.26 × 0.28 × 0.30 mm		
Crystal system	orthorhombic		
Space group	$P2_12_12_1$ (No. 19)		
Unit cell parameters	a =7.2590(5) Å	α =90.00°	
	b =11.2161(8) Å	β =90.00°	
	c =20.2136(14) Å	γ = 90.00°	
	V = 1645.7(2) $Å^3$	Z = 4	F(000) = 832
Density (calcd)	1.696 g/cm^3		
Diffractometer	Bruker CCD		
Radiation	graphite-monochromatized Mo K_α, λ = 0.71073 Å		
Temperature	296±2K		
Scan type	ω-scan		
Data collection range	$-9 < h < 9$, $-13 < k < 14$, $-26 < l < 20$; $\theta_{max} = 27.7°$		
Reflections measured	Total: 9981	Unique (n): 3786	Observed [I ≥ 2σ(I)]: 3551
Absorption coefficient	1.971 mm^{-1}		
No. of variables, p	204		
Weighting scheme	$w = \frac{1}{\sigma^2(F_o^2) + (0.001P)^2 + 1.0P}$		$P = (F_o^2 + 2F_c^2)/3$
$R1 = \frac{\Sigma \|\|F_o\| - \|F_c\|\|}{\Sigma \|F_o\|}$ (for all reflections)	0.0324	0.0297 (for observed data)	
$wR2 = \sqrt{\frac{\Sigma[w(F_o^2 - F_c^2)^2]}{\Sigma w(F_o^2)^2}}$ (for all reflections)	0.0625	0.0617 (for observed data)	
Goof = S = $\sqrt{\frac{\Sigma[w(F_o^2 - F_c^2)^2]}{n - p}}$	1.215		
Largest and mean Δ/σ	0.000, 0.000		
Residual extrema in final difference map	-0.748 to 0.469 e $Å^{-3}$		

Table S2: Atomic coordinates and equivalent isotropic temperature factors* (Å^2)

Atoms	*x*	*y*	*z*	$U_{eq.}$
I(1)	0.68270(5)	0.07899(3)	0.13922(2)	0.04477(10)
O(1)	0.7238(4)	0.5965(3)	0.14053(18)	0.0449(8)
O(2)	0.9815(5)	0.6651(3)	0.08456(19)	0.0472(9)
O(3)	1.1558(5)	0.6083(3)	0.17089(16)	0.0390(8)
O(4)	1.0048(5)	0.4741(3)	0.27055(16)	0.0414(8)
O(5)	0.5400(5)	0.3579(3)	0.15372(14)	0.0369(8)
O(6)	0.2859(5)	0.3247(3)	0.21333(18)	0.0505(10)
C(1)	0.8689(7)	0.6809(4)	0.1401(3)	0.0414(11)
C(2)	0.9919(7)	0.6544(4)	0.2000(3)	0.0359(11)
C(3)	0.8927(6)	0.5547(4)	0.2362(2)	0.0330(10)
C(4)	0.7783(6)	0.4981(4)	0.1811(2)	0.0324(10)
C(5)	1.1684(8)	0.6593(4)	0.1063(2)	0.0404(11)
C(6)	1.2758(7)	0.5759(5)	0.0623(3)	0.0529(13)
C(7)	1.2518(10)	0.7829(5)	0.1087(3)	0.071(2)
C(8)	0.6082(7)	0.4350(5)	0.2062(2)	0.0415(11)
C(9)	0.3796(6)	0.3043(4)	0.1659(2)	0.0318(10)
C(10)	0.3283(7)	0.2204(3)	0.1119(2)	0.0302(9)
C(11)	0.4378(6)	0.1271(4)	0.0906(2)	0.0307(9)
C(12)	0.3826(7)	0.0556(4)	0.0376(2)	0.0392(11)
C(13)	0.2153(8)	0.0769(5)	0.0070(2)	0.0492(13)
C(14)	0.1007(8)	0.1671(5)	0.0288(3)	0.0499(14)
C(15)	0.1553(7)	0.2377(4)	0.0818(3)	0.0406(11)

*$U_{eq.}$ defined as one third of the trace of the orthogonalized **U** tensor.

Table S3: Bond lengths (Å) and bond angles (°)

I(1)-C(11)	2.101(4)	C(2)-C(3)	1.517(6)
O(1)-C(1)	1.417(5)	C(3)-C(4)	1.527(6)
O(1)-C(4)	1.431(5)	C(4)-C(8)	1.511(6)
O(2)-C(1)	1.401(6)	C(5)-C(6)	1.508(7)
O(2)-C(5)	1.428(7)	C(5)-C(7)	1.513(7)
O(3)-C(2)	1.424(6)	C(9)-C(10)	1.488(6)
O(3)-C(5)	1.428(5)	C(10)-C(11)	1.383(6)
O(4)-C(3)	1.401(6)	C(10)-C(15)	1.409(7)
O(5)-C(9)	1.333(5)	C(11)-C(12)	1.398(6)
O(5)-C(8)	1.455(5)	C(12)-C(13)	1.384(7)
O(6)-C(9)	1.198(5)	C(13)-C(14)	1.382(8)
C(1)-C(2)	1.534(7)	C(14)-C(15)	1.389(7)
C(1)-O(1)-C(4)	108.3(3)	O(3)-C(5)-C(6)	108.9(4)
C(1)-O(2)-C(5)	108.3(4)	O(2)-C(5)-C(7)	110.4(5)
C(2)-O(3)-C(5)	106.6(4)	O(3)-C(5)-C(7)	111.3(4)
C(9)-O(5)-C(8)	115.5(3)	C(6)-C(5)-C(7)	112.4(5)
O(2)-C(1)-O(1)	110.7(4)	O(6)-C(9)-O(5)	123.9(4)
O(2)-C(1)-C(2)	105.6(4)	O(6)-C(9)-C(10)	124.5(4)
O(1)-C(1)-C(2)	107.4(4)	O(5)-C(9)-C(10)	111.6(4)
O(3)-C(2)-C(3)	109.2(4)	C(11)-C(10)-C(15)	118.8(4)
O(3)-C(2)-C(1)	103.3(4)	C(11)-C(10)-C(9)	124.2(4)
C(3)-C(2)-C(1)	104.3(4)	C(15)-C(10)-C(9)	116.9(4)
O(4)-C(3)-C(2)	116.0(4)	C(10)-C(11)-C(12)	120.5(4)
O(4)-C(3)-C(4)	114.2(4)	C(10)-C(11)-I(1)	122.3(3)
C(2)-C(3)-C(4)	102.4(4)	C(12)-C(11)-I(1)	117.0(3)
O(1)-C(4)-C(8)	109.1(4)	C(13)-C(12)-C(11)	119.7(5)
O(1)-C(4)-C(3)	104.3(3)	C(14)-C(13)-C(12)	120.8(5)
C(8)-C(4)-C(3)	113.2(4)	C(13)-C(14)-C(15)	119.5(5)
O(2)-C(5)-O(3)	103.8(4)	C(14)-C(15)-C(10)	120.6(5)
O(2)-C(5)-C(6)	109.7(4)		
Hydrogen bonding			
H(4)···O(6)$^{\#}$	2.22(6)	O(4)-H(4)···O(6)$^{\#1}$	153(6)

Symmetry transformations code: #1 (1+*x*, *y*, *z*).

Table S4: Anisotropic thermal parameters* (Å^2)

Atoms	U_{11}	U_{22}	U_{33}	U_{23}	U_{13}	U_{12}
I(1)	0.03459(14)	0.04980(16)	0.04992(17)	0.00413(17)	-0.00501(16)	0.00588(15)
O(1)	0.0325(17)	0.0482(18)	0.0541(18)	0.0055(19)	-0.0073(16)	-0.0039(14)
O(2)	0.0370(19)	0.058(2)	0.046(2)	0.0067(18)	-0.0011(18)	0.0021(18)
O(3)	0.0292(17)	0.0455(18)	0.0424(17)	0.0042(14)	0.0014(15)	-0.0024(15)
O(4)	0.039(2)	0.048(2)	0.0364(19)	0.0005(16)	0.0004(16)	-0.0006(16)
O(5)	0.0337(16)	0.0440(16)	0.0329(17)	-0.0114(14)	0.0065(14)	-0.0163(14)
O(6)	0.045(2)	0.0510(19)	0.056(2)	-0.0137(17)	0.0224(19)	-0.0146(17)
C(1)	0.040(3)	0.028(2)	0.056(3)	0.000(2)	0.002(3)	0.0043(17)
C(2)	0.036(3)	0.027(2)	0.045(3)	-0.009(2)	-0.001(2)	-0.0041(19)
C(3)	0.031(2)	0.032(2)	0.036(2)	-0.0090(18)	0.0014(19)	-0.0022(17)
C(4)	0.030(3)	0.032(2)	0.036(2)	-0.0079(18)	0.0019(19)	-0.0040(18)
C(5)	0.036(3)	0.036(2)	0.048(3)	0.004(2)	0.000(3)	-0.009(2)
C(6)	0.044(3)	0.058(3)	0.056(3)	-0.004(3)	0.007(2)	0.002(3)
C(7)	0.086(5)	0.049(3)	0.078(4)	0.001(3)	0.012(4)	-0.033(3)
C(8)	0.038(2)	0.048(3)	0.038(2)	-0.015(2)	0.005(2)	-0.015(2)
C(9)	0.030(2)	0.029(2)	0.037(2)	0.0012(18)	0.003(2)	-0.0035(17)
C(10)	0.027(2)	0.0294(19)	0.034(2)	0.0045(17)	0.000(2)	-0.009(2)
C(11)	0.028(2)	0.0277(19)	0.037(2)	0.0079(18)	0.001(2)	-0.0030(17)
C(12)	0.050(3)	0.034(2)	0.035(2)	-0.0010(19)	0.003(2)	-0.008(2)
C(13)	0.062(4)	0.053(3)	0.033(2)	0.005(2)	-0.013(2)	-0.023(3)
C(14)	0.039(3)	0.060(3)	0.051(3)	0.017(3)	-0.017(3)	-0.013(3)
C(15)	0.033(3)	0.036(2)	0.053(3)	0.006(2)	-0.005(2)	-0.002(2)

*The exponent takes the form: $-2\pi^2\Sigma\Sigma U_{ij}h_ih_j\mathbf{a}_i^*\mathbf{a}_j^*$

Table S5: Coordinates and isotropic temperature factors* (Å^2) for H atoms

Atoms	*x*	*y*	*z*	$U_{eq.}$
H(4)	1.069(9)	0.451(5)	0.246(3)	0.050
H(1)	0.8207	0.7625	0.1420	0.050
H(2)	1.0139	0.7245	0.2279	0.043
H(3)	0.8076	0.5910	0.2680	0.040
H(4A)	0.8545	0.4427	0.1554	0.039
H(6A)	1.3967	0.5642	0.0804	0.079
H(6B)	1.2857	0.6097	0.0189	0.079
H(6C)	1.2132	0.5006	0.0598	0.079
H(7A)	1.1751	0.8341	0.1349	0.107
H(7B)	1.2609	0.8142	0.0646	0.107
H(7C)	1.3724	0.7787	0.1280	0.107
H(8A)	0.6381	0.3880	0.2450	0.050
H(8B)	0.5147	0.4928	0.2183	0.050
H(12)	0.4580	-0.0060	0.0229	0.047
H(13)	0.1794	0.0299	-0.0287	0.059
H(14)	-0.0119	0.1805	0.0082	0.060
H(15)	0.0770	0.2968	0.0975	0.049

*The exponent takes the form: $-8\pi^2 U \sin^2\theta/\lambda^2$
